# Modulation of Tumor Metabolism in Acute Leukemia by Plant-Derived Polymolecular Drugs and Their Effects on Mitochondrial Function

**DOI:** 10.3390/molecules30081783

**Published:** 2025-04-16

**Authors:** Cindy Arévalo, Carolina Carlosama, Laura Rojas, Mónica P. Cala, Marie-Paule Hamon, Bertrand Friguet, Alfonso Barreto, Susana Fiorentino

**Affiliations:** 1 Grupo de Inmunobiología y Biología Celular, Facultad de Ciencias, Pontificia Universidad Javeriana, Bogotá 110231, Colombia; cindy.arevalo@javeriana.edu.co (C.A.); carolina_carlosamap@javeriana.edu.co (C.C.); rojasl.a@javeriana.edu.co (L.R.); alfonso.barreto@javeriana.edu.co (A.B.); 2 Centro de Metabolómica-MetCore, Universidad de los Andes, Bogotá 111711, Colombia; mp.cala10@uniandes.edu.co; 3 Institut de Biologie Paris-Seine, Sorbonne Université, Biological Adaptation and Ageing, 75005 Paris, France; mariepaulehamon@gmail.com (M.-P.H.); bertrand.friguet@upmc.fr (B.F.)

**Keywords:** acute leukemia, plant-derived polymolecular drugs, chemotherapy, metabolism, mitocans

## Abstract

The analysis of tumor metabolism offers promising opportunities for developing new therapeutic strategies. Plant-derived polymolecular drugs can regulate cellular metabolism, making them potential candidates for treatment. This study examined the metabolic effects of plant-derived polymolecular drugs—P2Et, Anamu-SC, and Esperanza—on leukemic cell lines (lymphoid and myeloid types) and primary leukemic blasts. The metabolic analysis included oxidative status, glucose consumption, extracellular acidification, oxygen consumption, mitochondrial dynamics, and untargeted metabolomics. Additionally, the effect of co-treatment with conventional chemotherapeutic drugs was investigated. Results showed that P2Et and Anamu-SC reduced the viability and proliferation of all tumor cell lines, exhibiting antioxidant effects. Anamu-SC decreased reactive oxygen species levels in lymphoid tumor cells. Mitochondrial activity was selectively affected by the plant-derived polymolecular drugs, with Anamu-SC and Esperanza causing more significant, potentially reversible damage compared to P2Et. Anamu-SC and Esperanza increased levels of phosphatidylcholines and carnitines. The co-administration of plant-derived polymolecular drugs with chemotherapeutics improved the cytostatic efficacy of cytarabine. In conclusion, this research highlights the promising pharmacological activity of Anamu-SC and Esperanza as mitocans for the treatment of acute leukemia. The study emphasizes the practical significance of combining plant-derived polymolecular drugs with conventional chemotherapeutics to enhance their cytostatic efficacy.

## 1. Introduction

Acute leukemia (AL) is neoplasms characterized by the clonal proliferation and abnormal differentiation of hematopoietic stem cells or progenitor cells of myeloid and/or lymphoid origin [1]. Leukemia cells utilize several nutrition sources to obtain energy and biomass, enabling them to develop metabolic adaptability to life and proliferate [2]. A high rate of the pentose pathway has been found in acute lymphoid leukemia (ALL) cells, allowing them to generate the nucleotides and antioxidants necessary for their survival. They also have a greater metabolic rate and employ amino acids—especially asparagine—and glycolysis to produce ATP [3]. On the other hand, myeloid leukemia cells (AML) consume fatty acids, glucose, and glutamine [2,4] and have unique mitochondrial alterations that decrease their respiratory capacity, making them more sensitive to therapies that induce oxidative stress [5].

Plant-derived polymolecular drugs (PDPDs) are standardized complex mixtures from plants that can engage multiple targets, exhibiting synergistic biological effects [6]. Research on PDPDs as an alternative and/or adjuvant therapy in cancer in conjunction with chemotherapy has increased significantly in the last ten years in the field of integrative medicine, seeking to evaluate the safety, efficacy, and economic impact of PDPDs on patient survival [7,8,9]. It was shown that the overall survival rate is higher in pediatric and adult leukemia patients who use Traditional Chinese Medicine (TCM) compared to non-users. TCM as an adjuvant therapy combined with Western Medicine, can alleviate adverse symptoms, improve quality of life, and prolong overall survival without incurring excessive medical expenses [10]. The positive impacts of PDPDs combined with chemotherapeutic drugs seem to be linked to decreasing chemoresistance, changing the tumor microenvironment, and triggering various signaling pathways related to cell death, cell growth, and tumor metabolism, as well as boosting the immune system [11,12,13].

There is substantial evidence supporting the traditional use of *Caesalpinia spinosa* (*C. spinosa*) [7] and *Petiveria alliacea* (*P. alliacea*) [14] to treat a variety of diseases. The seeds and pods of *C. spinosa* have demonstrated antibacterial, antitumor, astringent, anti-inflammatory, and wound-healing properties [15]. The genus *Caesalpinia* has shown effects on AL, with the dried heartwood of the *Caesalpinia sappan* species being prescribed in traditional Chinese medicine to treat AML [16], and was found to possess an antiproliferative capacity and induce apoptosis in leukemic cells in vitro [17]. Ethnobotanical data show that *P. alliacea* exhibits a wide range of activities, including anxiolytic, antidepressant, antinociceptive, and anticonvulsant properties, as well as cognitive enhancement [14]. The roots and leaves of *P. alliacea* have been used in folk medicine due to their antispasmodic, sedative, diuretic, anthelmintic, analgesic, and anti-inflammatory effects [18]. Regarding its influence on metabolism, traditional knowledge attributes hypoglycemic properties to it; however, previous research generated contradictory results [19,20]. In cancer, aqueous infusions have been used in the treatment of leukemia and breast cancer [21].

Our research group demonstrated the anticancer effects of P2Et obtained from *C. spinosa* and the Anamu-SC extract obtained from *P. alliacea* on leukemia, melanoma, and breast cancer models [8,22,23]. Among P2Et’s biological actions are the ability to induce apoptosis and caspase activation, reduction in PgP-type (P-glycoprotein 1) resistance pump action [24], induction of immunogenic cell death [23,25], and promotion of multifunctional T lymphocyte activation [26], while exhibiting antioxidant properties [27]. The modification of glycolytic pathway enzymes, which results in a decrease in lactate generation and the reduction in β-F1-ATPase expression, seems to be the mechanism underlying the anticancer activity of Anamu-SC [28]. In addition, we recently described that primary myeloid blasts obtained from AL patients are more susceptible to the Anamu-SC extract than lymphoid blasts, suggesting different patterns common to lineage differences [29].

Exploring whether the effectiveness of PDPDs against various tumor types is linked to the underlying metabolic differences among tumor cells is essential. By examining the effects of P2Et and Anamu-SC on metabolism, we aimed to determine if the energy usage patterns of lymphoid and myeloid cells influence their response to common chemotherapy drugs for AL. The PDPDs alter mitochondrial function differently in primary blasts and cell lines, impacting cell growth. When combined with chemotherapy, they can selectively enhance or reduce its effectiveness, indicating that, while PDPDs show potential, their use must be rigorously evaluated.

## 2. Results

### 2.1. Myeloid Cells Produce Fewer ROS, Consume More Glucose, and Duplicate at a Faster Rate than Lymphoid Cells

**Figure 1:**
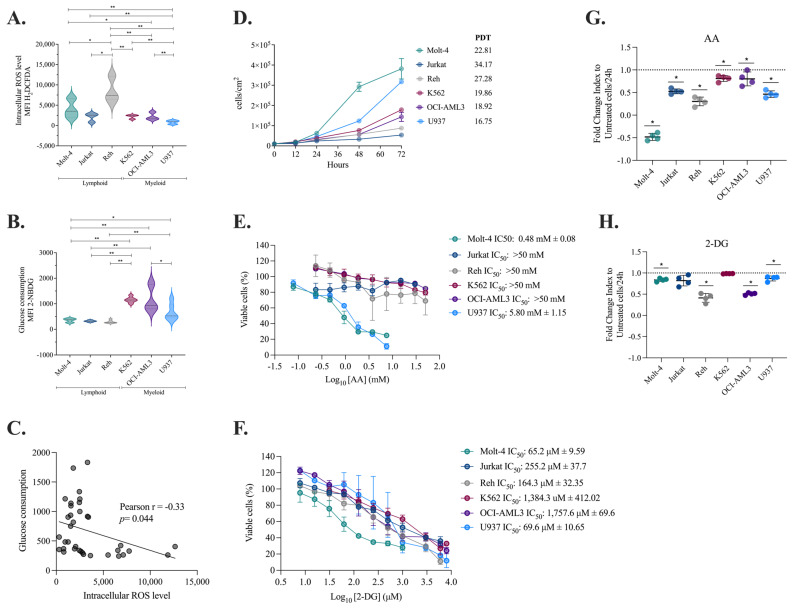
Metabolic differences between lymphoid and myeloid leukemia cells and their response to metabolic modulators. (**A**) Comparative analysis of intracellular ROS levels in lymphoid and myeloid leukemia cell lines at baseline conditions (Molt-4-MFI: 3522 ± 2.151, Jurkat-MFI: 2540 ± 994.6, Reh-MFI: 7441 ± 2943, K562-MFI: 2459 ± 478.7, OCI-AML3-MFI: 1809 ± 861.7, U937-MFI: 946 ± 432.6). (**B**) Comparative analysis of glucose consumption in lymphoid and myeloid leukemia cell lines at baseline conditions (Molt-4-MFI: 374 ± 75, Jurkat-MFI: 318 ± 33.1, Reh-MFI: 261 ± 61.8, K562-MFI: 1145 ± 112.7, OCI-AML3-MFI: 925.5 ± 485.2, U937-MFI: 524.5 ± 326). (**C**) Pearson correlation analysis between glucose consumption and ROS levels. (**D**) Comparison of PDTs between lymphoid and myeloid cell lines. (**E**) Cytotoxicity evaluations in leukemic cells treated with AA. (**F**) Cytotoxicity evaluations in leukemic cells treated with 2-DG. (**G**) Fold change in the leukemia cells’ proliferation index 24 h after they were treated with AA. (**H**) Fold change in the leukemia cells’ proliferation index 24 h after they were treated with 2-DG. The data are represented as the mean ± SD for two or three independent experiments. * *p* < 0.05; ** *p* < 0.01. AA: ascorbic acid, 2-DG: 2-deoxy-d-glucose.

Changing bioenergetics, increasing biosynthesis, and redox balance are some of the most common reprogramming activities that help leukemia cells survive [30]. New research has shown that metabolic changes are different depending on the type and subtype of tumor and the tumor microenvironment [31]. Considering the possibility that this metabolic plasticity influences its proliferative capacity and is related to the exogenous agent response, we wished to assess these variables. The basal metabolic characterization of the cell lines showed that myeloid cells produce fewer ROS (Figure 1A) and consume more glucose (Figure 1B) than lymphoid cell lines revealing an inverse relationship between ROS production and glucose consumption (Figure 1C).

Subsequently, the relationship between metabolic characteristics and cell proliferation potential was investigated (Figure 1D). It was observed that lymphoid cells replicate in 22 to 27 h, while myeloid cells replicate in 16 to 19 h, resulting in a 1.51-fold higher proliferation rate. These data suggest that the origin and/or maturational stage of tumor cells impacts their proliferative potential and that, as expected, increased glucose intake is associated with a proliferative advantage [32,33].

To assess the impact of metabolic regulation on cell viability and proliferation, we treated six leukemia cell lines with ascorbic acid (AA), a potent antioxidant, and 2-deoxyglucose (2-DG), a glycolysis inhibitor. The selection of these compounds was based on previous findings showing that two of our PDPDs modulate energy and oxidative metabolism: P2Et exhibits antioxidant activity, while extracts derived from *P. alliacea* alter glucose metabolism [27,28]. The inclusion of AA and 2-DG as controls provided a functional reference, allowing for a more precise characterization and comparison of the metabolic effects induced by our extracts in subsequent studies. In terms of viability, we found that Molt-4 and U937 were especially prone to AA activity, while Jurkat, Reh, K562, and OCI-AML3 were less susceptible (Figure 1E; Table S2). Regarding 2-DG treatment, although a reduction in the viability rate was evidenced, no lineage-dependent response pattern was observed, but as expected, the glycolytic myeloid lines K562 and OCI-AML3 required extremely high doses of 2-DG to induce cell death (Figure 1F, Table S2) compared to the other cell lines. The high sensitivity of U937 cells to AA is maybe because these cells may require ROS for survival, and reducing them even in small amounts reduces their chances of proliferation. Furthermore, the susceptibility of MOLT-4 cells to 2-DG may depend not only on their basal glucose consumption but also on their dependence on glycolysis and their limited ability to compensate for its inhibition through alternative metabolic pathways.

An analysis of the proliferative capacity modulation induced by AA or 2-DG revealed that exposure to AA reduced the proliferation of all six cell lines but was particularly noticeable in lymphoid cells (Figure 1G; Table S3), suggesting that ROS might play a role in promoting proliferation in these cells, as previously published [34]. As anticipated, 2-DG treatment similarly decreased the ability of Molt-4, Reh, OCI-AML3, and U937 cells to divide (Figure 1H, Table S3), albeit to a lesser extent.

These results suggest that basal metabolic differences between lymphoid and myeloid leukemias influence their proliferation but do not determine a differential response to AA and 2-DG. Indeed, sensitivity to these inhibitors may be modulated by other factors, such as the intrinsic antioxidant capacity of each cell line or metabolic plasticity. Notably, the glycolytic myeloid lines K562 and OCI-AML3 required higher doses of 2-DG to induce cell death, supporting their greater dependence on glycolysis. These findings highlight the complexity of metabolic regulation in leukemias and the need for combined approaches for their therapeutic modulation.

### 2.2. Decreased Intracellular ROS Levels Affect the Viability and Proliferation of Myeloid and Lymphoid Leukemia Cells

**Figure 2:**
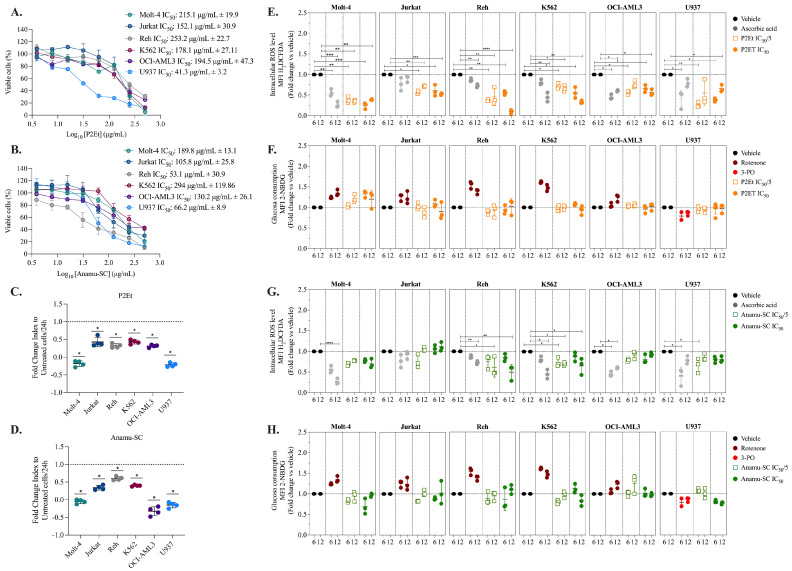
Evaluation of the anti-leukemic activity of P2Et and Anamu-SC. (**A**) Cytotoxicity evaluations in leukemic cells treated with P2Et. (**B**) Cytotoxicity evaluations in leukemic cells treated with Anamu-SC. (**C**) Fold change in the leukemia cells’ proliferation index 24 h after they were treated with P2Et. (**D**) Fold change in the leukemia cells’ proliferation index 24 h after they were treated with Anamu-SC. (**E**) Fold change in H_2_DCFDA MFI after treatments with IC_50_ and IC_50_/5 of P2Et extract, 2 mM Ara-C (positive control), doxorubicin IC_50_ (positive control), or ascorbic acid (negative control) for 6 h and 12 h in all leukemic cells. (**F**) Fold change in 2-NBDG MFI after treatments with IC_50_ and IC_50_/5 of P2Et extract, Rotenone (positive control), or 3-PO (negative control) for 6 h and 12 h in all leukemic cells. (**G**) Fold change in H_2_DCFDA MFI after treatments with IC_50_ and IC_50_/5 of Anamu-SC extract, 2 mM Ara-C (positive control), doxorubicin IC_50_ (positive control), or ascorbic acid (negative control) for 6 h and 12 h in all leukemic cells. (**H**) Fold change in 2-NBDG MFI after treatments with IC_50_ and IC_50_/5 of Anamu-SC extract, Rotenone (positive control), or 3-PO (negative control) for 6 h and 12 h in all leukemic cells. Data are represented as the mean ± SD for two or three independent experiments; * *p* < 0.05; ** *p* < 0.01, *** *p* < 0.001, **** *p* < 0.0001. AA: ascorbic acid.

Considering the antitumor activity of P2Et and Anamu-SC, previously studied by our group, their effect on cell viability and the proliferation of these same lines was evaluated. P2Et was found to be cytotoxic to all six cell lines at high concentrations, with less impact on Reh and Molt-4 (Figure 2A). The Anamu-SC exhibited stronger cytotoxic activity on Reh and U937 (Figure 2B, Table S2). Furthermore, after treatment with the two PDPDs, we observed a significant decrease in proliferation in all lines (Figure 2C,D, Table S3). As a result, P2Et and Anamu-SC demonstrated both cytotoxic and antiproliferative actions in myeloid and lymphoid leukemia cell lines.

ROS levels and glucose consumption were then evaluated after treatment with each extract. P2Et reduced intracellular ROS starting at 6 h in several cell lines, both at IC_50_ and at sublethal concentrations (Figure 2E), but did not appear to exert any activity on glucose uptake (Figure 2F). On the other hand, Anamu-SC shared the antioxidant properties of P2Et (Figure 2G), but, in contrast, tended to decrease glucose consumption (Figure 2H) compared to P2Et, mainly on K562 and U937 cells. These findings suggest that while AA and 2-DG differentially modulate cell viability and proliferation, P2Et and Anamu-SC exhibit a broader and more consistent effect across both types of leukemia. This highlights their potential as anti-leukemic agents with multiple mechanisms of action beyond cellular metabolism.

### 2.3. P2Et and Anamu-SC Are Intracellular Antioxidants in Patient-Derived Leukemia Blasts

**Figure 3:**
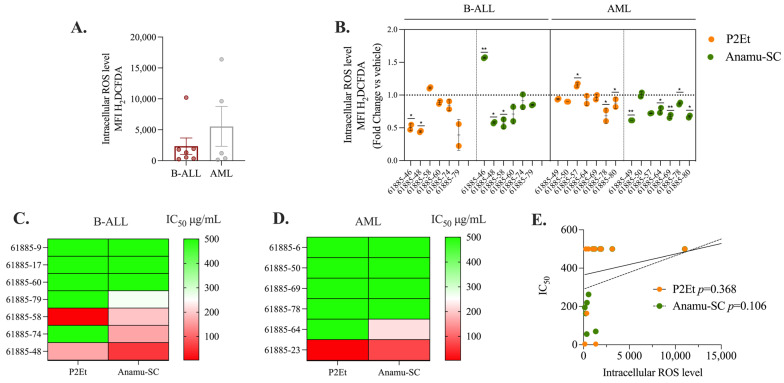
P2Et and Anamu-SC extracts’ anti-leukemia effects on patient-derived leukemia blasts. (**A**) Intracellular ROS levels in leukemic blasts (B-ALL-MFI: 1144 ± 671.9; AML-MFI: 4661.8 ± 6452). (**B**) Measurement of intracellular ROS in leukemic blasts after 6 h of treatment with P2Et and Anamu-SC. (**C**) Response of blasts derived from B-ALL patients to the extracts. (**D**) Response of blasts derived from AML patients to the extracts. Red color indicates higher sensitivity, and green color indicates lower sensitivity. Sensitivity is based on calculated IC_50_ values. (**E**) Pearson correlation analysis between the IC_50_ of P2Et or Anamu-SC extracts and basal intracellular ROS levels. The data are shown as the mean of the two measured replicates ± SD; * *p* < 0.05; ** *p* < 0.01.

Modulation of the intra- and extracellular oxidative microenvironment may have implications in the response to therapy. In leukemias, both pro-oxidant and antioxidant activity can help control the disease [35]. The standardized P2Et and Anamu-SC can reduce intracellular ROS levels in cell lines, so we studied if this effect is also exerted in the primary leukemia blasts of AL patients at the time of diagnosis. To conduct a preliminary evaluation, we included seven patients with B-ALL and five with AML. No significant differences in basal ROS levels were detected between the B-ALL and AML blasts (Figure 3A). However, within the AML patient group, two samples (61885-49 and 61885-50) exhibited an increase in ROS levels compared to the rest of the group. After P2Et and Anamu-SC treatment, ROS levels were significantly reduced in both myeloid and B-lymphoid blasts (Figure 3B) except in a patient with B-ALL (61885-46), where Anamu-SC acted as a pro-oxidant agent. This finding confirmed the antioxidant capacity of P2Et and Anamu-SC in leukemia blasts, as previously found on cell lines, but made evident the individual specificities of each one. The cytotoxic activity of P2Et and Anamu-SC was then tested in patients with B-ALL and AML blasts. Overall, leukemic blasts appeared to be more susceptible to Anamu-SC than to P2Et, particularly in patients with lymphoid leukemia (Figure 3C,D). However, no relationship with basal ROS levels was found (Figure 3E). These results, in line with our previous studies [29], further support the potential of the antioxidants P2Et and Anamu-SC in modulating the oxidative metabolism of leukemic cells. Their ability to regulate intracellular ROS levels and exert cytotoxic effects underscores their relevance as potential therapeutic agents. However, the observed variability in response among patients highlights the importance of considering individual metabolic profiles to optimize treatment strategies and enhance therapeutic efficacy.

### 2.4. P2Et and Anamu-SC Modulate Energy Metabolism and Affect Mitochondrial Function

**Figure 4:**
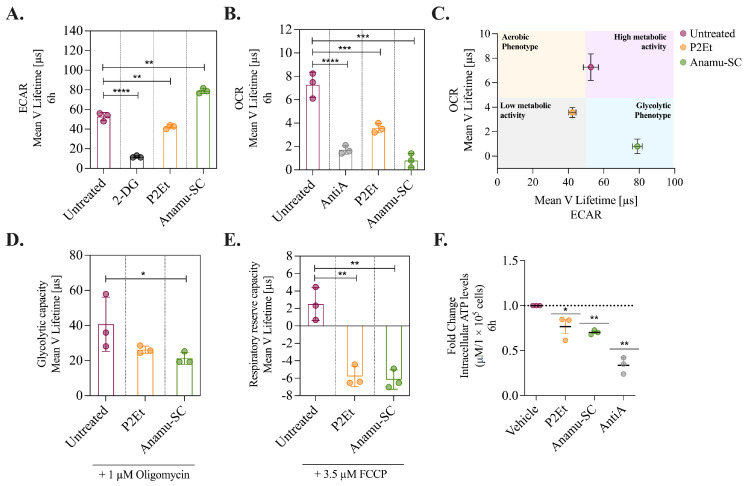
Changes in energy metabolism in K562 myeloid cells caused by P2Et and Anamu-SC. (**A**) ECAR and (**B**) OCR without or with 6 h treatment with P2Et and Anamu-SC. (**C**) Comparative metabolic map of the bioenergetic phenotype. (**D**) Glycolytic capacity of cells without or with pretreatment with the extracts and then exposed to 1 µmol oligomycin for 2 h. (**E**) Respiratory reserve capacity of cells without or with pretreatment with the extracts and then exposed to 3.5 µmol FCCP for 1.5 h. (**F**) Intracellular ATP levels post-treatment for 6 h with the extracts. The data are represented as the mean ± SD for three independent experiments; * *p* < 0.05; ** *p* < 0.01, *** *p* < 0.001, **** *p* < 0.0001. OCR: oxygen consumption rate, ECAR: extracellular acidification rate, 2-DG: 2-deoxy-d-glucose, AntiA: Antimycin A.

The participation of energy metabolism modulation in the activity of the extracts was evaluated. Mitochondria plays an important role in energy production, and it was reported that the K562 myeloid cells have a higher number of mitochondria and mitochondrial activity than other myeloid lines [36], making it a good model for evaluating the activity of various compounds on mitochondria. Then, K562 cells also carry the *BCR-ABL1* oncogene [37,38], resembling an aggressive AML. The oxygen consumption rate (OCR) and the extracellular acidification rate (ECAR) were measured before and after treatment with P2Et and Anamu-SC. OCR indicates mitochondrial respiration, while ECAR serves as an indirect indicator of glycolysis. As inhibitory controls, we used AntiA for OCR and 2-DG for ECAR. The administration of Anamu-SC resulted in a minor increase in extracellular acidification, while P2Et was found to partially reduce it (Figure 4A). Interestingly, both extracts substantially reduced oxygen consumption (Figure 4B). K562 cells under basal conditions exhibited a high metabolic state, according to their OCR and ECAR. P2Et caused a decrease in mitochondrial respiration and glycolytic activity, leading to a shift to a low metabolic state, but Anamu-SC principally reduced mitochondrial activity (Figure 4C).

To determine whether the metabolic changes were transient or permanent, K562 cells were treated with P2Et and Anamu-SC, and with oligomycin or FCCP. Oligomycin inhibits ATP synthase in the ETC, decreasing the ATP/ADP ratio and driving glycolysis [39]. The difference in ECAR before and after oligomycin addition is equal to the glycolytic capacity of the cells. Cells exposed to oligomycin exhibited a glycolytic capacity of 40.6 ± 15.4 Lifetime (μs) and, interestingly, pretreatment with P2Et or Anamu-SC resulted in a decrease of 18% (33.2 ± 0.68 Lifetime (μs)) and 47% (21.2 ± 3.4 Lifetime (μs)), respectively (Figure 4D).

Regarding mitochondrial activity, the impact of the P2Et and Anamu-SC on the respiratory reserve of the cells was evaluated. The cells were treated with the uncoupler FCCP, which caused a strong increase in oxygen consumption. The difference between basal respiration and maximum respiration after FCCP treatment represented the respiratory reserve capacity of the cells. K562 cells have a low respiratory reserve capacity of only 2.4 ± 1.8 Lifetime (μs) (Figure 4E), which is consistent with previous publications [6]. Both P2Et and Anamu-SC markedly decreased the respiratory reserve capacity, reaching negative values (Figure 4E). As expected, both extracts induced an early drop in intracellular ATP levels, as did the AntiA (Figure 4F). These findings show that P2Et and Anamu-SC can alter mitochondrial function in leukemic cells and confirm previous findings in breast cancer cells with a different extract of *P. alliacea* [40].

### 2.5. Anamu-SC but Not P2Et Increases Mitochondrial Fragmentation and Induces Changes in the Mitochondrial Membrane Potential (ΔΨm) of K562 Myeloid Cells

Considering that fission and fusion events may have an impact on mitochondrial function [41], we explored whether the extracts caused changes in the K562 mitochondrial morphology. We used AA and 2-DG as controls, and both these controls and the extracts were combined with FCCP. Morphometric parameters extracted from confocal microscopy images included the number of mitochondrial particles per cell (Nc), the mean area of each mitochondrial particle (Am), mitochondrial mass (calculated by multiplying Nc by Am), a measure of mitochondrial length (AR), a combined measure of mitochondrial length and degree of branching (FF), and, finally, mitochondrial fragmentation.

**Figure 5:**
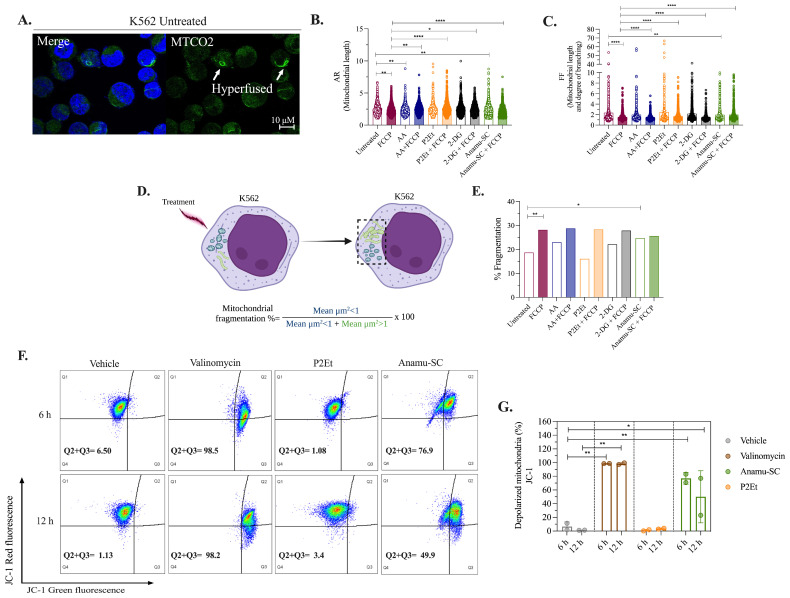
Morphometric and mitochondrial depolarization analysis in K562 myeloid cells. (**A**) Images captured by confocal microscopy of cells labeled with MTCO2 (green) and DAPI (blue). Parameters extracted from images captured by confocal microscopy under basal and post-treatment conditions with the metabolic modulators and/or FCCP 3.5 µmol. (**B**) AR. (**C**) FF. (**D**) Schematic showing mitochondrial fragmentation analysis. Cells were treated (left). The dotted box (right) marks the mitochondria that responded in the injured cell. The degree of fragmentation was calculated by dividing the area of injured cells with fragmented mitochondria (blue) by the area of cells containing responding mitochondria (dotted box) [43]. (**E**) Percentage of fragmentation under basal conditions (left axis); percentage of cell death (right axis). (**F**) Analysis of ΔΨm using the JC-1 probe by flow cytometry, upper panel at 6 h and lower panel at 12 h. (**G**) Percentage of cells with mitochondrial depolarization. Data are represented as the mean ± SD for two independent experiments; * *p* < 0.05; ** *p* < 0.01, **** *p* < 0.0001.

A significant proportion of K562 cells showed fragmented and sporadically hyperfused mitochondria under basal conditions (Figure 5A). Among the parameters evaluated, AR, FF, and mitochondrial fragmentation were the most affected (Figure 5), while the other parameters showed less impact (Figure S1). FCCP alone decreased AR and FF and induced mitochondrial fragmentation (Figure 5B–E). AA increased mitochondrial length (Figure 5B), but this effect was lost when combined with FCCP. In contrast, P2Et, both alone and in combination with FCCP, appeared to increase mitochondrial length (Figure 5B) and was accompanied by an increase in intermediate-sized mitochondria (Figure S1E) without inducing mitochondrial fragmentation. Regarding 2-DG, no significant changes were observed in the mitochondrial network, either alone or in combination. For its part, Anamu-SC significantly reduced AR (Figure 5B) and FF (Figure 5C) and induced mitochondrial fragmentation (Figure 5D,E), maintaining this effect when combined with FCCP.

After, we assessed the effect of P2Et and Anamu-SC on ΔΨm, a crucial factor in energy storage during oxidative phosphorylation (OxPhos) and, therefore, a global indicator of mitochondrial function [42]. The extracts were administered to K562 myeloid cells for 6 and 12 h (Figure 5F,G). Valinomycin, employed as a positive control, produced depolarization in more than 80% of the cells in both assessments. P2Et did not cause depolarization of the ΔΨm, whereas Anamu-SC did after 6 h. Taken together, our findings indicate that, while both P2Et and Anamu-SC affect mitochondrial function, the latter achieves this through a mechanism that alters mitochondrial dynamics and disrupts processes involved in the regulation of ΔΨm.

### 2.6. Induction of Apoptosis and Tumor Selectivity of P2Et and P. alliacea Extracts

**Figure 6:**
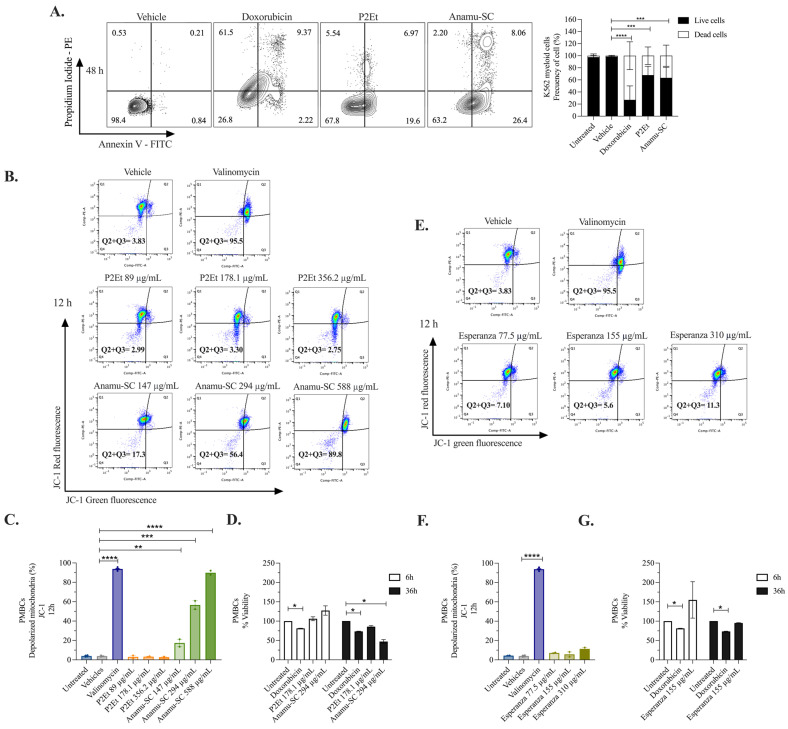
Evaluation of viability and depolarization of the mitochondrial membrane in PBMCs treated with P2Et and extracts derived from *P. alliacea*. (**A**) Cell death induction and frequency of live cells and dead cells in K562 myeloid cells by flow cytometry. (**B**) Analysis of ΔΨm using the JC-1 probe by flow cytometry in PMBCs treated with P2Et and Anamu-SC for 12 h. (**C**) Percentage of PMBCs with mitochondrial depolarization after 12 h of treatment with P2Et and Anamu-SC. (**D**) Percentage of viable PMBCs after 6 h and 36 h of treatment with P2Et and Anamu-SC. (**E**) Analysis of ΔΨm using the JC-1 probe by flow cytometry in PMBCs treated with Esperanza for 12 h. (**F**) Percentage of PMBCs with mitochondrial depolarization after 12 h of treatment with Esperanza. (**G**) Percentage of viable PMBCs after 6 and 36 h of treatment with Esperanza. The data from two independent experiments are presented. The *p*-values indicate a statistically significant difference between the compared treatments; * *p* < 0.05, ** *p* < 0.01, *** *p* < 0.001, **** *p* < 0.0001.

To evaluate whether activity on mitochondria induced apoptosis, we measured the changes by double staining with annexin V-FITC/PI. As shown in Figure 6A, the frequency of dead K562 cells (including those in early apoptosis, late apoptosis, and necrosis) was 73.11% after 48 h of treatment with doxorubicin. In comparison, P2Et induced 32.15% cell death, while Anamu-SC resulted in 36.72%. To determine whether the activity was specific to tumor cells, stimulated human peripheral blood mononuclear cells (PBMCs) from healthy donors were treated with phytohemagglutinin. P2Et did not affect ΔΨm; however, Anamu-SC caused a dose-dependent alteration of ΔΨm after 12 h (Figure 6B,C). Regarding viability, PBMCs remained viable after 36 h of stimulation with phytohemagglutinin and 6 h of treatment with the extracts. However, after 36 h of treatment, cell viability dropped by 14.4% in P2Et-treated cells and 52.9% in Anamu-SC-treated cells, as indicated in Figure 6D.

The Anamu-SC extract contains sulfur compounds, which have been linked to *P. alliacea*’s biological activity; however, the development of a phytotherapeutic product enriched in this metabolite has been hindered due to its high toxicity. For this reason, we recently developed an aqueous extract from *P. alliacea* named Esperanza, which was earlier studied. Esperanza is significantly richer in primary metabolites and has a lower content of sulfur compounds. This extract reduces the cell proliferation rate without inducing apoptosis and reduces the OCR consumption and the intracellular ATP, together with some metabolic changes suggesting the disruption of mitochondrial function [22,44]. Esperanza influences mitochondrial dynamics (Figure S2) and causes alterations in energy metabolism like those experienced with Anamu-SC [44]. Despite that, it does not alter the ΔΨm (Figure 6E,F) or viability (Figure 6G) of PBMCs at different concentrations, which may mitigate a harmful effect on normal cells if taken in vivo. The selectivity index has also been calculated for P2Et (Figure S3); for Esperanza, it was published in [44] using other normal cells, 3T3 and HpdLF. These observations reinforce the importance of evaluating the specificity of PDPDs on both tumor and normal cells and highlight Esperanza as a potential candidate for developing less cytotoxic therapies while still impacting tumor metabolic reprogramming.

### 2.7. Anamu-SC Induces Alterations in Glycerophospholipids, Fatty Acids, and Purine Nucleosides in K562 Cells

After observing what appears to be irreversible mitochondrial injury induced by *P. alliacea*’s extracts, we performed an untargeted metabolomic analysis on cell lysates from K562 cells treated with the Anamu-SC extract for 12 h. The results obtained were compared with previously published data on the metabolic changes induced by Esperanza in the same cell line [44]. The OPLS-DA model (Figure S4A) demonstrated a clear distinction between cell lysates treated with the Anamu-SC extract (green dots) and those treated with ethanol (blue dots), with R^2^ values of 0.740 and Q^2^ values of 0.981. By using a combination of MVA with variable importance in projection (VIP > 1 with JK) and UVA (*p* < 0.05), a total of 17 altered metabolites were identified in the cell lysates treated with Anamu-SC. Table S4 and the heat map (Figure S4B) reveal that cells treated with the Anamu-SC extract exhibited elevated levels of metabolites including indoleacrylic acid, glycerophospholipids such as phosphocholines (PC) (18:3), fatty acyls like hydroxylinoleoylcarnitine and hydroxyoctadecatrienoylcarnitine, purine nucleosides (adenosine, disodium adenosine phosphate, and adenosine monophosphate), and organoheterocyclic compounds such as biotin. Conversely, reduced levels of other metabolites (shown in the blue color range) were observed, including indoles and derivatives such as tryptophanol, glycerophospholipids like glyceryl phosphorylcholine and PIP-18:0, nucleosides, and nucleotides such as hydroxyguanosine, fatty acyls like propionyl carnitine and pentanamide, carboxylic acids such as cysteinyl glycine and glutathione, and organooxygen compounds like pantothenate. After identifying the metabolites altered by Anamu-SC, a comparison was made with the metabolites modified by Esperanza [44]. It was observed that the extracts obtained from *P. alliacea* shared some altered metabolites, inducing their increase or decrease, except for glutathione and adenosine monophosphate, which were affected differently (Table 1). These results indicate that both *P. alliacea* extracts induced significant alterations in biochemical metabolites in K562 cells, particularly in PC (18:3), and in metabolites involved in mitochondrial biochemical processes such as carnitines.

### 2.8. Regulation of Energy Metabolism Can Positively or Negatively Interfere with Chemotherapeutics

PDPDs, as well as metabolic modulators, can have synergistic, additive, or antagonistic effects with chemotherapy [45,46]. We, therefore, evaluated the effect on cell viability of our PDPDs with chemotherapeutics used in the treatment of AL. First, we found the IC_50_s of IDA and Ara-C chemotherapeutics on myeloid cells (Figure S5; right panel) and MTX, VIN, and DOX on lymphoid lines (Figure S5; left panel). Although it was impossible to calculate the IC_50_ values for MTX and VIN on the Molt-4 and Jurkat cell lines, standard concentrations of 10 µmol and 10 nM, respectively, were utilized for the interaction testing. Similarly, Ara-C only had a cytotoxic effect on the U937 cell line, so a concentration of 2 mM was used to assess the combinatorial effect. All leukemia lines were treated with 1.25 mM of AA.

**Figure 7:**
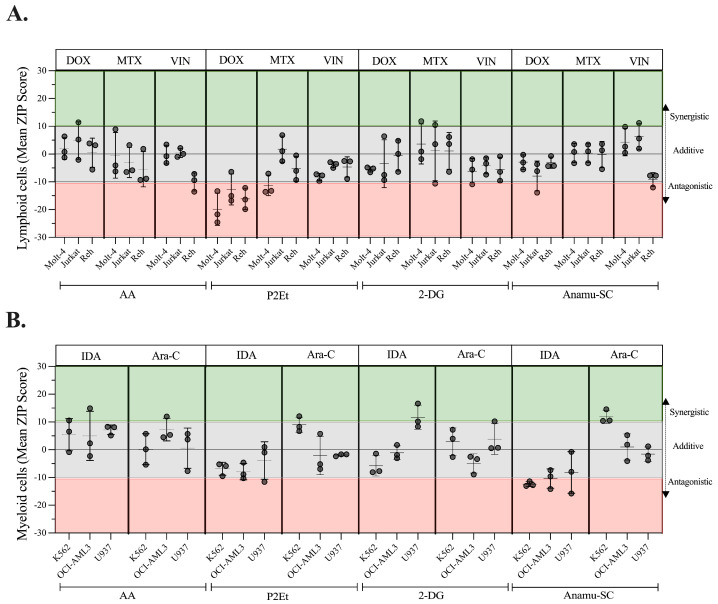
Summary of ZIP scores obtained from the combination of metabolic modulators and chemotherapeutics on lymphoid and myeloid leukemia cells. (**A**) Combinations of AA, P2Et, 2-DG, or Anamu-SC with DOX, MTX, and VIN on lymphoid cells. (**B**) Combinations of AA, P2Et, 2-DG, or Anamu-SC with IDA and Ara-C on myeloid cells. Green area, synergistic values; gray area, additive values; red area, antagonistic values. Data are represented as the mean ± SD for three independent experiments.

A ZIP score above 10 was interpreted as a synergistic effect, while a score below 10 was considered antagonistic. A value between −10 and 10 was classified as additive [47]. The results are summarized in Table S5. Regarding the lymphoid lines (Figure 7A), AA combined with DOX, MTX, or VIN showed an additive effect on all three lines, as did 2-DG and Anamu-SC. With P2Et, the interaction with DOX presented an antagonistic effect on the three lines, while the interaction with VIN was additive. On the Molt-4 line, P2Et and MTX were antagonistic. On myeloid cells (Figure 7B), AA and P2Et presented an additive effect combined with IDA and Ara-C; however, 2-DG combined with IDA as well as Anamu-SC with Ara-C showed a synergistic effect on lines U937 and K562, respectively.

As a result, at the concentrations tested, the use of antioxidants such as AA did not increase or alter the activity of some conventional chemotherapeutics, whereas the combination of P2Et and DOX was antagonistic in our model. The interaction of 2-DG or Anamu-SC with drugs seemed to have the same (additive) effect on lymphoid cells, while each of them acted differently on myeloid cells. This suggests that additional factors such as cell lineage or genetic changes, among others, may influence the response to combined treatments.

K562 and U937 cells, despite being myeloid in origin, respond differently to all treatments in terms of cytotoxicity and PDT rates, highlighting the heterogeneity of AML. The viability of the U937 line is significantly reduced when glucose consumption is inhibited with 2-DG in addition to IDA’s pro-oxidant activity, confirming the benefit of choosing two different targets (one of which was metabolic) for AL treatment. Although K562 was the least sensitive to Anamu-SC, it was interesting that, when combined with Ara-C, it converted the drug’s cytostatic effect to cytotoxic. By allowing the treatments to act for 24 h and then combining them with the chemotherapy for another 12 h, the impact of Anamu-SC and P2Et on Ara-C or IDA in K562 cells was further demonstrated (Figure S6). At the same time, the synergistic effect was also evident when combining Ara-C and Esperanza (ZIP synergy score: 20, 51) (Figure S7). If the lines are viewed as individual leukemia models, it is suggested that the individual sensitivity of patient-derived leukemia blasts to combination therapies be assessed before initiating the respective chemotherapy.

## 3. Discussion

Tumor metabolism has gained increasing attention due to its potential as a therapeutic target for both synthetic drugs and plant-derived extracts, which offer promising sources of phytomedicines. However, there is a lack of studies addressing the mechanistic aspects of the anti-leukemic effects of natural products through metabolic modulation, particularly when combined with current chemotherapeutic agents. To address this gap, this study evaluated the metabolic differences between lymphoid and myeloid cells, as well as their biological and metabolic responses to three natural extracts: P2Et, Anamu-SC, and Esperanza. One of the main limitations of this study is the limited number of fresh samples available, which restricted the ability to thoroughly evaluate the ex vivo sensitivity of leukemic blasts to phytomedicines. This limitation may affect the representativeness of the results and highlight the need for a larger sample size to validate the observations. Another significant limitation is the evaluation of the potentiation of Ara-C activity by *P. alliacea*. Although the preliminary results are promising, further studies combining it with other cytostatics and in more complex biological models are essential to confirm its efficacy. Finally, although mitochondria are proposed as a key target for these phytomedicines, the lack of detailed mechanistic studies limits the full understanding of their therapeutic potential in other tumor models. This highlights the need for further research to fully explore the underlying mechanisms involved in mitochondrial modulation as a target for natural antitumor therapies and the mechanisms underlying selectivity over normal cells.

Metabolic alterations in leukemia cells contribute to their growth and progression [48]. Efforts have been made to clarify the common and differential metabolic needs between leukemia subtypes [49,50], and several metabolomics studies have found differential circulating metabolites between patients with ALL and AML [51,52]. Citrate, choline, and betaine levels are significantly higher in patients with AML than in those with ALL [53,54], and differences in the lipid level between the two leukemias have also been identified [52]. Our research confirms that lymphoid cells exhibit distinct metabolic characteristics compared to myeloid cells in terms of intracellular ROS levels and glucose consumption. These two elements have been thoroughly investigated as a crucial component in clarifying the efficacy of different drugs [55,56]. In fact, differences in the intracellular oxidation degree have been reported between different myeloid lines [56,57]. On the other hand, previous reports of basal metabolic characterization (considering OCR vs. ECAR) of 19 lines, including Molt-4, Jurkat, Reh, and K562, did not show a lineage-associated pattern. However, Molt-4 cells presented a higher glycolytic capacity, followed by Jurkat, K562, and Reh [58]. In our work, basal metabolic characterization performed on primary cells derived from patients let us show a higher glycolytic pattern in AML primary blast compared to those from patients with common B phenotype ALL, following the same pattern as that observed in cell lines. Our results show that the oxidation degree inside lymphoid cells is greater, and this finding can be explained by their reduced antioxidant capacity. The Mn-SOD (mitochondrial superoxide dismutase 2), an antioxidant enzyme, is reduced in ALL and overregulated in AML [59]. Also, the intracellular content of GSH (an antioxidant tripeptide) in myeloid leukemias tends to be higher than in lymphoid leukemias [60].

The analysis of cell lines’ differences also evidenced the proliferative advantage of myeloid cells over lymphoid cells. Although AML and ALL share alterations in signaling pathways that promote their proliferation, such as Wnt/β-catenin, PI3K/AKT/mTOR, NOTCH, and JAK/STAT [61,62], AML cells are characterized by high molecular heterogeneity that may favor the upregulation of other metabolic pathways such as NF-κB, Hedgehog, EGFR, TGF/SMAD, and PPAR contributing to their high replication rate [61,62]. All these findings highlight the importance of understanding metabolic differences between leukemia subtypes for the development of more precise and effective therapeutic approaches. For example, it was observed that lymphoid leukemic cells with a low rate of glycolytic activity could be more susceptible to L-asparaginase treatments [58] while myeloid lines with a high level of glycolysis, driven by mutations in FLT3-ITD, could show a greater response to melatonin [63].

The cytotoxic and antiproliferative effects on tumor cells of plant-derived extracts and isolated compounds [64,65] and their impact on the regulation of tumor metabolism have been widely investigated [11,57]. In the context of leukemias, both glycolysis and OxPhos were identified as possible therapeutic targets, although the scientific literature presents contradictory results in this regard. Some studies suggest that leukemic cells could reprogram their metabolism after OxPhos inhibition but not after glycolysis inhibition, indicating that suppression of the glycolytic pathway could be a more effective therapeutic strategy [66]. However, more recent research revealed that acute leukemias, especially the myeloid subtype, show a vulnerability to mitochondrial damage [5,67,68,69], which seems to be a preferential target of some natural products. The methanol extract of *Uvaria longipes* showed cytotoxic and antiproliferative effects, mainly in hepatocellular carcinoma [70]. Fermented wheat germ extract has anti-leukemia activity on the Jurkat cell line through the inhibition of glucose-6-phosphate dehydrogenase (G6PDH) and transketolase enzymes, charging to regulate carbon flux in the glycolytic and pentose pathway, respectively [71]. Lipid B extracted from avocado exerts a selective cytotoxic effect on AML leukemia cells because it can enter the mitochondria through carnitine palmitoyltransferase I (CPT1), an enzyme that facilitates the transport of mitochondrial lipids, favoring their accumulation and, consequently, the inhibition of fatty acid oxidation and a reduction in NADPH levels, leading to cell death [72]. Some other studies showed that other polyphenols, such as emodin, quercetin, and cis-stilbene, have a greater effect on lymphoid than on myeloid cell lines [55,73]. However, the underlying antitumor mechanisms involving metabolic regulation in leukemia cells have been little explored, and even more so if complex extracts are involved.

In this work, we show that extracts of P2Et, obtained from *C. spinosa*, and Anamu-SC and Esperanza, obtained from *P. alliacea*, affect leukemic metabolism. This modulation may delay proliferation and thus have lethal effects on the myeloid line K562. The main components of the P2Et extract are gallic acid and various gallates. Despite its diverse biological activities as an antioxidant [74] or inhibitor of mitochondrial respiration through the deregulation of the Akt/mTOR signaling pathway [75], the use of isolated gallic acid has been limited because of its poor bioavailability, low stability, and absorption. Our observations indicate that, on the tumor cells, P2Et decreases both ECAR and OCR along with respiratory reserve capacity and ATP synthesis. Despite these effects, there does not appear to be any change in ΔΨm or mitochondrial dynamics attributable to P2Et. This suggests that P2Et could alter other cellular functions, which would subsequently affect the mitochondria. Previously, in melanoma studies, it was shown that P2Et can activate PERK, promote calcium release from the reticulum, and trigger the upregulation of immunogenic cell death markers.

Anamu-SC extract is characterized by the presence of sulfur, sterols, triterpenes, and phenolic compounds such as flavonoids [29]. The influence of triterpenoids on tumor metabolism has been the subject of studies due to their ability to inhibit the enzyme fatty acid synthase, responsible for long-chain fatty acids’ synthesis as well as the reduction in the glycolytic enzyme pyruvate kinase 2 [76]. In addition, phenolic compounds were shown to affect mitochondria by interacting with electron transport chain complexes, I and III, and ATP synthase as well as altering membrane potential by acting as protonophores [77]. Investigations carried out by our group evidenced an antiglycolytic activity of *P. alliacea*, evidenced in breast cancer cells [28]. In addition, we recently demonstrated that Esperanza extract, obtained from *P. alliacea*, is enriched in primary metabolites such as glycolic acid, which induces apoptosis by activating caspase-3 in leukemic cells [78], and quinic acid, which exhibits antioxidant capacity and decreases angiogenesis in breast cancer cells [79].

**Figure 8:**
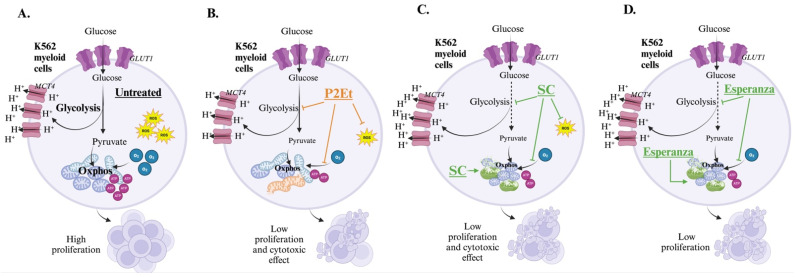
Model of the metabolic regulation of P2Et and *P. alliacea* on leukemic cells. (**A**) The K562 myeloid cells have high metabolic activity and are primarily dependent on OxPhos and glycolysis, which support sustained proliferation. (**B**) P2Et-treated K562 cells exhibit reduced metabolic activity with indirect mitochondrial damage, resulting in their low proliferation and death. (**C**) Anamu-SC-treated K562 myeloid cells have significant mitochondrial damage that leads them to depend on glycolysis to survive; however, it appears to be ineffective glycolysis, which is why the cells reduce their proliferation and death. (**D**) Esperanza-treated K562 myeloid cells have significant mitochondrial damage that leads them to depend on glycolysis to survive; however, it appears to be ineffective glycolysis, which is why the cells reduce their proliferation.

Both Anamu-SC and Esperanza extracts from *P. alliacea* show a slight increase in ECAR, traditionally interpreted as an increase in the glycolytic pathway. However, nowadays, it is known that extracellular acidification can be induced by alternative mechanisms [80]. Our results showed that *P. alliacea* extracts could induce significant metabolic stress evidenced by ΔΨm dysfunction, decreased OCR, the early reduction in ATP production, increased mitochondrial fragmentation, as well as alteration of metabolites such as PC (18:3) and carnitines. Mitochondria contain double membranes, which are composed mainly of phospholipids. Although mitochondria can synthesize some phospholipids, most of them, like PCs, are biosynthesized in the endoplasmic reticulum. Contacts between the endoplasmic reticulum and mitochondria are required for the transfer of certain phospholipids that are essential for mitochondrial phospholipid metabolism, membrane structure, and function. Any change in these processes can affect energy production in the mitochondria and contribute to metabolic disorders [81,82]. Studies have shown that the accumulation of carnitines, in the context of mitochondrial dysfunction, can disturb fatty acid metabolism and energy production, contributing to various metabolic disorders [83,84]. This finding supports the idea that *P. alliacea* extracts induce a strong irreversible mitochondrial injury, resulting in cell death. In this work, it was discovered that purifying Anamu-SC using supercritical fluids appears to concentrate extremely toxic metabolites such as DTS and DBS, hence increasing the unwanted harmful effects on normal cells. In contrast, the aqueous extract (Esperanza) preserves its anti-leukemic effect by modulating tumor metabolism without damaging normal cells. Figure 8 depicts a model of the metabolic changes generated by the extracts examined in K562 cells. Interestingly, it was shown that the induced mitochondrial alterations that can generate danger signals were associated with increased tumor immunogenicity and the activation of a more effective immune response in cancer [85].

At the in vitro level, we identified that the interaction between P2Et extract and DOX in leukemic lymphoid cells was antagonistic. Our hypothesis regarding this result is based on the differences in the main biological activity of each treatment, particularly in its influence on intracellular oxidation. On the one hand, the phenolic compounds present in P2Et are characterized by their potent antioxidant activity, suggesting that they could neutralize DOX-induced ROS. These ROS are essential for the induction of apoptosis in tumor cells [86,87]. Furthermore, the effect of P2Et on mitochondrial function could interfere with the participation of mitochondria in the redox cycle of the quinone group of DOX, affecting the generation of ROS. In addition to the antagonistic effect, we observed a synergistic effect between Ara-C and SC, which can be explained because they are treatments with different action targets. On the one hand, Ara-C, a cytosine analogue, inhibits DNA synthesis, while SC modulates ROS and alters mitochondria, the cellular cytoskeleton, and glycolytic metabolism [28,88]. This combination significantly affects the regenerative capacity of K562 cells, leading to a greater induction of cell death. Other studies have also reported antagonistic and synergistic effects to natural products. A lipid-rich avocado seed extract (Mexican native) can enhance the cytostatic activity of cisplatin in the osteosarcoma model [89]. DOX and etoposide in combination with polyphenols synergistically reduced ATP levels, induced apoptosis, and increased cell cycle arrest in the S and/or G2/M phases of lymphoid leukemia cell lines. However, in myeloid cell lines, the effects of the combination treatments varied; quercetin, apigenin, emodin, and cis-stilbene had a synergistic or additive effect when combined with DOX, but an antagonistic effect was observed when combined with rhein [90]. Additionally, the green tea polyphenol epigallocatechin-3-gallate in combination with ATRA supports the degradation of the PML/RARα oncogene, restoring PML and inducing tumor promyelocyte differentiation [91]. The combination of the hypoglycemic compound, buformin, with ascorbate, an antioxidant, markedly affects the mitochondrial activity of primary AML cells [56]. Likewise, diphenyl-iodonium (DPI), an inhibitor of NADPH-oxidase, potentiates Ara-C activity [92]. P2Et exhibits enormous intracellular antioxidant activity; however, this does not seem to be responsible for all its biological functions. When AA was used as a control, different effects than those observed for P2Et were evident in the synergy test. The same was observed for Anamu-SC, Esperanza, and 2-DG, suggesting that the main activity of Anamu-SC extracts is on mitochondrial function. In summary, the synergy between PDPDs and chemotherapeutics offers a promising avenue to improve cancer treatment, increase the efficacy of drugs, and reduce their side effects. However, more clinical research is required to validate these findings and establish clear guidelines for their combined use. Anamu-SC, Esperanza, and 2-DG, in turn, have been associated with increased tumor immunogenicity and the activation of a more effective immune response in cancer.

## 4. Materials and Methods

### 4.1. Cell Culture and Treatment

The cells used were purchased from ATCC (American Type Culture Collection, Manassas, VA, USA) or as a donation from the Institute Biology Paris Seine (Sorbonne Université, CNRS, INSERM, Paris, France). Human leukemia cells of myeloid lineages K562, OCI-AML3, and U937 and lymphoid lineages Molt-4, Jurkat, and Reh were routinely cultured at a density of 5 × 10^5^ cells/mL for exponential growth in a medium containing RPMI-1640, (GIBCO^®^, Waltham, MA, USA) with l-glutamine (2 mM, Eurobio^®^, Les Ulis, France), HEPES (10 mM, Eurobio^®^, France), sodium pyruvate (1 mM, Eurobio^®^, France), Fetal Bovine Serum (FBS, 10% Eurobio^®^, France), and Penicillin/Streptomycin (1% Corning^®^, Corning, NY, USA) at 37 °C and 5% CO_2_. Additionally, their morphology was verified by microscopy, and the assays were performed on cultures with >95% viability. Doxorubicin (DOX), methotrexate (MTX), Vincristine (VIN), idarubicin (IDA), Ara-C, (3-(3-Pyridinyl)-1-(4-pyridinyl)-2-propen-1-one) (3PO), oligomycin, and antimycin A (AntiA) were diluted in dimethyl sulfoxide (DMSO). Ascorbic acid (AA) and 2-deoxy-d-glucose (2-DG) were diluted in H_2_O, while carbonylcyanide-p-trifluoromethoxyphenylhydrazone (FCCP) and rotenone were diluted in absolute ethanol.

### 4.2. PDPDs Obtained from C. spinosa and P. alliacea

The research was authorized by the Colombian Environmental Ministry agreement for Access to Genetic Resources and Derived Products No. 220/2018 (RGE 0287-6). *Caesalpinia spinosa (Molina) Kuntze* plant pods were collected in Villa de Leyva, Boyacá, Colombia, and identified by Luis Carlos Jimenez from the Colombian National Herbarium (voucher specimen number COL 523714). P2Et was produced under good manufacturing practice (GMP). It was chemically characterized and standardized as previously described [24,93]. In each assay, the dry extract of P2Et was diluted in 95% ethanol, obtaining a fresh solution of 50 mg/mL, or in a 95% ethanol/H_2_O 1:1 ratio for the MitoXpress and pH-Xtra assays. For the Anamu-SC, plant material was identified by Antonio Luis Mejia from the Colombian National Herbarium (voucher number COL 333406). Anamu-SC was produced from the leaves of *Petiveria alliacea* L., as we described before [29].

The stock solution of P2Et and Anamu-SC was prepared by reconstitution in 95% ethanol at a concentration of 50 mg/mL or in a 95% ethanol/H_2_O 1:1 ratio for the MitoXpress and pH-Xtra assays and stored at 4 °C. The aqueous extract of *P. alliacea*, called Esperanza, was obtained and previously characterized by our group, as reported [22]. The production of Esperanza was carried out under good manufacturing practice (GMP) conditions at LabFarve Laboratories with subsequent physicochemical and microbiological certification before starting the experiment.

### 4.3. Patients and Peripheral Blood-Derived Leukemia Blasts

Patients with AL diagnosed and treated at the Hematology Service at the Hospital Universitario San Ignacio (HUSI), between 2019 and 2023, were included in this study. Informed consent of all patients in the study was obtained according to the Declaration of Helsinki, and the studies were approved by the ethics committee of Centro Javeriano de Oncología, Colombia (decision number #2019/062). A total of 17 patients were recruited (9 men and 8 women; median age 53.8 years, with a range of 21–76 years). Patient details are given in Table S1. Bone marrow and peripheral blood samples were collected. Mononuclear cells were isolated by density gradient centrifugation over Ficoll-Plaque (Sigma, St. Louis, MO, USA) according to the manufacturer’s protocol. The patients were diagnosed and classified according to the hospital’s clinical guidelines considering the immunophenotype results evaluated by flow cytometry, the morphological evaluation in the myelogram, the genetic alterations studied by conventional karyotype and/or molecular biology, and the clinical characteristics recorded in the histories.

### 4.4. Intracellular ROS Measurement

To evaluate the production of reactive oxygen species (ROS) in the cell lines, 2.5 × 10^5^ cells were seeded in 12-well plates and treated with IC_50_ and IC_50_/5 of P2Et and Anamu-SC extracts for 6 h and 12 h. Ascorbic acid (5 mM) for 2 h was used as an antioxidant control, and DMSO or ethanol was used as negative controls (0.02%). The protocol described previously [94] was followed. Briefly, cells were stained with 1 µmol of 2′,7′ dichlorodihydrofluorescein diacetate (H2DCFDA) (Sigma Aldrich, Saint Louis, MO, USA) for 40 min at 37 °C, followed by propidium iodide (PI) (Sigma-Aldrich, St. Louis, MO, USA). Each sample was then acquired using a FACSAria IIu (BD, Franklin Lakes, NJ, USA) and analyzed with FlowJo v10.8.1 software (BD Life Sciences, Franklin Lakes, NJ, USA). Experiments were performed in duplicate on three independent experiments and the results were expressed as mean fluorescence intensity (MFI) ± SEM.

In leukemic blasts derived from AL patients from bone marrow whole blood, ROS levels were measured before and after treatment with the two extracts at a concentration of 50 μg/mL for 6 h. Briefly, the sample was diluted 1/20 in RPMI 1640 without phenol red. One mL per well was added in 48 plates and it was labeled with the H_2_DCFDA probe at a concentration of 120 µmol for 30 min at 37 °C in the dark [95]. Then, the labeling was performed with the CD45 APC/Cy7 antibody (BioLegend Cat. 368516, BioLegend, San Diego, CA, USA) using a 1/10 dilution and left to incubate for 15 min at room temperature in the dark. Subsequently, ammonium chloride 1× was added for 15 min at room temperature in the dark. Two washes with PBS 1× were performed and PI was added. It was immediately read on the FACSAria IIu (BD) flow cytometer and analyzed with FlowJo v10.8.1 software (BD Life Sciences).

### 4.5. Glucose Consumption Assay

For the evaluation of glucose uptake in the cell lines, 2.5 × 10^5^ cells were seeded in 12-well plates and treated with IC_50_ and IC_50_/5 of P2Et and Anamu-SC extracts for 6 h and 12 h. Rotenone (200 nM) was used as an inductor control, 3PO (40 µmol) was used as an inhibitor control, and DMSO or ethanol was used as negative controls (0.02%). Based on the description in [94], the cells were resuspended in 50 µmol of 2-NBDG [2-(*N*-(7-Nitrobenz-2-oxa-1,3-diazol-4-yl)amino)-2-deoxyglucose] (Cayman Chemicals, Ann Arbor, MI, USA), which was prepared in PBS 1×. Then, the cells were incubated for 30 min at 37 °C and washed with cold PBS 1×; the PI was added just before starting the reading on the FACSAria IIu flow cytometer (BD) and analyzed with the FlowJo v10.8.1 software (BD Life Sciences). Experiments were performed in duplicate on two or three independent experiments and the results were expressed as MFI ± SEM.

### 4.6. In Vitro Cytotoxicity Assay

The cytotoxic effects of P2Et, Anamu-SC, DOX, MTX, and VIN on Molt-4, Jurkat, and Reh and of Anamu-SC, P2Et, IDA, and Ara-C on K562, OCI-A3, and U937 were evaluated using 3-(4,5-dimethylthiazol-2-yl)-2,5-diphenyltetrazolium bromide (MTT) (Sigma-Aldrich), as previously reported [88]. Cells (6 × 10^4^ cells/well) were seeded in 96-well plates with DMSO, ethanol, and/or H_2_O (0.02%) as negative controls. After 48 h of the treatments, the IC_50_ (50% inhibition of cell growth) value was calculated using a non-linear regression log (inhibitor) versus a response–variable slope graph in GraphPad Prism version 10.0.0 for Mac OS X statistics software (GraphPad Software, San Diego, CA, USA).

To evaluate the cytotoxic activity of P2Et, Anamu-SC, and Esperanza on normal cells, 1 × 10^5^ PBMCs isolated from healthy individuals were seeded in 96-well, U-bottom plates. Subsequently, these cells were stimulated for 36 h with phytohemagglutinin (5 µg/mL) before being treated with three concentrations of the extracts. Three different concentrations were used: 89 µg/, 178.1 µg/mL, and 356.2 µg/mL for P2Et; 147 µg/mL, 294 µg/mL, and 588 µg/mL for Anamu-SC; and 77.5 µg/mL, 155 µg/mL, and 310 µg/mL for Esperanza.

### 4.7. Proliferation Assay

Leukemia cells were seeded in 6-well plates at a density of 10,638 cells/cm^2^ (100,000 cells/mL) and treated with the IC_50_ of P2Et, Anamu-SC, 2-DG, and 1.25 mM of AA. After 12, 24, 48, and 72 h, cells were collected and counted with 0.4% trypan blue. Using the exponential growth method (Malthusian), the population doubling time (PDT) was calculated through GraphPad Prism version 10.0.0 for Mac OS X statistics software (GraphPad Software, San Diego, CA, USA), described before [44]. The proliferation index after the treatments of each line was calculated using the formula: Log_10_(*N*/*N*0) × 3.33, where *N* = the number of cells at the time of harvesting and *N*0 = is the number of cells plated [96].

### 4.8. Oxygen Consumption and Extracellular Acidification Rates

The Agilent MitoXpress (Agilent, Santa Clara, CA, USA) and pH-Xtra assays were used to measure the oxygen consumption rate (OCR) and the extracellular acidification rate (ECAR) in the Cytation 5 Reader (BioTek, Winooski, VT, USA), following the manufacturer’s instructions, as described before [44,97]. ECAR and OCR were simultaneously measured in basal conditions and after the subsequent treatment. Briefly, K562 myeloid cells were plated at a density of 1.25 × 10^5^ cells in a 96-well plate and then treated with the IC_50_ of P2Et or Anamu-SC for 6 h. Before carrying out the measurement, the controls were added. As ECAR controls, 50 mM 2-DG and 1 μM oligomycin were used, and for the OCR, 3.5 μM FCCP and 1 μM AntiA were used. Dual-read TR-F and subsequent Lifetime calculation allow measurement of the rate of fluorescence decay of MitoXpress Xtra and pH-Xtra reagents. The dual intensity readings were used to calculate the corresponding Lifetime (µs) using the following transformation: Lifetime (µs) [τ] = (D2 − D1)/ln(IW1/IW2), where IW1/and IW2 represent the two (dual) measurement windows and D1 and D2 represent the delay time before the measurement of W1 and W2, respectively.

### 4.9. Mitochondrial Morphological Analysis

For the immunofluorescent labeling of mitochondria, 2.5 × 10^5^ K562 myeloid cells were seeded in 12-well plates in 1 mL of RPMI-1640 medium (Thermo Fisher Scientific, Waltham, MA, USA). Subsequently, they were treated for 6 h with the IC_50_ of P2Et, Anamu-SC, 2-DG, 155 μg/mL Esperanza, and 5 mM AA, without or with 3.5 μM FCCP for 30 min. Using [98] as a reference, at the end of time, 85 × 10^3^ cells were spread on round coverslips. Cells were fixed with 4% paraformaldehyde and 0.1% glutaraldehyde for 20 min, then washed and permeabilized with 0.1% Triton X-100 for 10 min. Next, blocking was carried out with 50 mM NH_4_Cl for 10 min, followed by incubation with a polyclonal antibody directed against subunit 2 of cytochrome c oxidase (Anti-MTCO2 Abcam ab110258, Abcam, Waltham, MA, USA) for 1.45 h and secondary anti-rabbit IgG (H + L) antibody Alexa Fluor^®^ 568 (Life Technologies A11004, Life Technologies, Woburn, MA, USA) for 50 min at room temperature in the dark. Finally, the cells were stained with DAPI (300 nM) for 5 min, and coverslips were mounted on slides with Prolong Antifade Reagent (Life Technologies, Woburn, MA, USA). Images were acquired with an Olympus FV1000 confocal microscope (Olympus, Tokyo, Japan), with a 60× PlanAPO oil objective; a multi-argon laser with a 488 nm line was used to visualize MTCO2 and a multi-iodine laser with a 405 nm line was used to visualize DAPI. Images were generated with X, Y, and Z dimensions (X: 640, 0.0–105.435 [μm], 0.165 [um/Pixel]; Y: 640, 0.0–105.435 [μm], 0.165 [μm/Pixel]; and Z: 9, 5002.41–5004.81 [μm], 0.3 [μm/Slice]). Quantitative mitochondrial analysis was performed on 40 cells per treatment (two independent experiments) on a Z projection according to the protocol described by Koopman et al. [99] and Tronstad et al. [100] using ImageJ2 software v.2.9.0/1.53t. Parameters calculated from the images for each condition were the number of mitochondrial objects per cell (Nc), the mean area of each mitochondrial object (Am), mitochondrial mass (the product of Nc and Am), the mean mitochondrial object aspect ratio ((AR) [(major axis)/(minor axis)], reflects the “length-to-width ratio”, a measure of mitochondrial length), and the mean mitochondrial object shape/form factor (FF) [(perimeter^2^)/(4π·surface area)], a combined measure of mitochondrial length and degree of branching. The following two types of subpopulation classification were defined: (I) size populations (small, μm^2^ < 1; intermedium, μm^2^ 1–5; larger, μm^2^ > 5) and (II) shape populations (spherical, *FF*  =  1–3; elongated, *FF*  =  3–4; filamentous, *FF*  >  4) [101]. The percentage of mitochondrial fragmentation was also calculated following what was described [43].

### 4.10. Cell Death Evaluation

Phosphatidylserine externalization was assessed using Annexin V-FITC/PI (Molecular Probes, Invitrogen Corp, Carlsbad, CA, USA)/PI (Sigma, Saint Louis, MO, USA), as previously reported [102]. A total of 2.5 × 10^5^ K562 myeloid cells were treated with the IC_50_ of P2Et or Anamu-SC extracts for 48 h at 37 °C. DMSO (<1%) or H_2_O was used as a negative control and doxorubicin (0.40 µmol) was used as a positive control. After incubation, the cells were washed with PBS 1× and labeled with annexin V (Molecular Probes, Invitrogen Corp, Carlsbad, CA, USA) and PI for 15 min at room temperature (Sigma, St. Louis, MO, USA). A total of 20,000 events were acquired on a FACSAria II-U flow cytometer (Becton Dickinson, BD, Franklin Lakes, NJ, USA). The results were subsequently analyzed using FlowJo v10.8.1 software (BD Life Sciences, Franklin Lakes, NJ, USA). Double-negative cells were considered intact, whereas double-positive cells were considered late apoptosis cells. Annexin V^+^/PI^−^ cells were presumably in early apoptosis, and the Annexin V^−^/PI^+^ were considered necrotic cells.

### 4.11. Mitochondrial Membrane Potential (ΔΨm) Assay

Experiments with the JC-1 probe were performed to measure mitochondrial depolarization, following the methodology outlined in [88]. Cells (2.5 × 10^5^) were incubated with 2.5 μg/mL JC-1 (Sigma-Aldrich) at 37 °C for 10 min and washed twice with PBS 1× before analysis with a FACSAria IIu cytometer (Becton, Dickinson and Company, Franklin Lakes, NJ, USA). At high concentrations, JC-1 monomers form aggregates. The monomers fluoresce green, and the aggregates fluoresce red. As a positive depolarization control, 1 μM valinomycin was added. A depolarization evaluation was carried out after 6 h and 12 h of IC_50_ treatment with Anamu-SC, P2Et, and 155 μg/mL Esperanza. The same culture and stimulation protocols as for the cytotoxic evaluation were carried out on normal cells, and three concentrations of each extract were also evaluated. Duplicate estimations were made, and the average was expressed as mean ± SEM in two independent experiments.

### 4.12. Measurement of Intracellular ATP

The manufacturer’s protocol for the ATP HS II bioluminescence assay kit (Roche, Mannheim, Germany) was followed to measure intracellular ATP, as previously detailed [44]. Briefly, 2.5 × 10^5^ K562 cells were seeded in 12-well plates and then treated with P2Et (IC_50_ 178.1 μg/mL), SC (IC_50_ 294 μg/mL), Esperanza (155 μg/mL), AntiA (1 μM, positive control), and DMSO or ethanol (negative controls, 0.02%) for 6 h. Subsequently, the cells were lysed and centrifuged at 10,000× *g* for 1 min at room temperature. The supernatant was collected and frozen at −80 °C for 24 h. In dark-bottom, 96-well plates, 50 µL of each supernatant was mixed with 50 µL of luciferase reagent, and the bioluminescent signal was read on a Cytation 5 Cell Imaging multimodal reader using Agilent BioTek Gen5 v3.14 software. ATP concentration was calculated from a log–log plot of data obtained from an ATP standard curve prepared at each reading. Experiments were performed in duplicate in three independent experiments, and the results (RUL/100,000 cells) were expressed as the fold change over vehicle ± SD.

### 4.13. Untargeted Metabolomics Analysis by LC-QTOF-MS

The K562 cells (1 × 10^6^) were treated for 12 h with Anamu-SC extract at 254 μg/mL, Esperanza extract at 155 μg/mL, and ethanol as a negative control. As described in [44], following the incubation period, the cells were frozen in liquid nitrogen, washed three times with PBS 1× at 4 °C, and kept at 80 °C until they were processed further. For every treatment, six separate biological replicates were assessed. A total of 5 µL of the samples were injected and analyzed by liquid chromatography (LC) coupled to mass spectrometry with a time-of-flight analyzer (QTOF/MS), using an Agilent technology 1260 LC system coupled to a Q-TOF 6545 time-of-flight mass spectrometer with electrospray ionization. A gradient was used that included acetonitrile (Phase B) and 0.1% (*v*/*v*) formic acid in Milli-Q water (Phase A), with a constant flow rate of 0.3 mL/min and a temperature of 40 °C. The elution gradient was programmed with the following specifications: 5% B for 10 min, 5–95% for 12 min, and 95% for 2 min. Finally, the equipment was reconditioned with 5% B for 5 min. Acquisition for full scan mass spectrometry was performed in positive electrospray ionization (ESI) mode, covering the range of 70 to 1100 *m*/*z*. For mass correction, two reference masses, *m*/*z* 121.0509 [C_5_H_4_N_4_ + H]^+^ and *m*/*z* 922.0098 [C_18_H_18_O_6_N_3_P_3_F_24_ + H]^+,^ were employed throughout the analysis. The lysate cells extracted from every sample were combined in equal quantities to create quality control (QC) samples. This QC was injected six times at the beginning of the run and after every six samples.

#### Data Processing, Analysis, and Annotation of Statistically Significant Molecular Features

The data were deconvolved, aligned, and integrated into Agilent MassHunter Profinder B.10.0 software using recursive and molecular feature extraction algorithms. The final set of data was then manually examined and revised based on reproducibility and presence, accounting for the metabolites that were found in all samples at 100% as well as those with a coefficient of variation in the QC that was less than 20%. Subsequently, univariate (UVA) using a nonparametric test (Mann–Whitney U test) with the Benjamini–Hochberg False Discovery Rate post hoc correction (FDR) and multivariate (MVA) with an unsupervised principal component analysis (PCA) and supervised orthogonal partial least squares discriminant analysis (OPLS-DA) were used. This set of analyses was used to identify molecular features meeting at least one of the following criteria: (1) UVA: *p*-value < 0.05 and (2) MVA: significant variance in projection (VIP) > 1. The statistically significant characteristics were determined by initially searching the CEU MASS MEDIATOR tool for the masses and comparing them with the different online databases: KEGG (https://www.genome.jp/kegg/kegg1.html, accessed on October 2022), Lipid MAPS (https://lipidMAPS.org, accessed on October 2022), METLIN (https://metlin.scripps.edu, accessed on October 2022), and HMBD (https://hmdb.ca, accessed on October 2022). The annotation process included the generation of the molecular formula considering the monoisotopic distribution, verification of adduct formation, retention times, and comparison of experimental MS/MS spectra with those obtained in different online databases. Additionally, automatic annotation was performed with MSDIAL v4.8 (https://systemsomicslab.github.io/compms/msdial/main.html, accessed on November 2022). The reporting of annotation levels was carried out according to the guidelines presented in the previous literature [103].

### 4.14. Interaction Assays Between Antitumor Drugs and PDPDs

To assess the potential synergy of drug pairs against leukemia cells, we built 7 × 7 or 7 × 6 treatment combination landscapes using the bioconductor package “synergy finder” (https://synergyfinder.org/#!/, accessed on November 2023) and its zero-interaction potency (ZIP) model [8]. For every landscape, we treated cells with serial 2-fold dilutions of treatment(s) from the calculated IC_50_ or base concentration (for AA, MTX, VIN, Ara-C, or Esperanza) (two concentrations above and four concentrations below). In the treatments for which the IC_50_ could not be calculated, an average concentration was used. We considered a treatment combination to be synergistic against leukemia cells when the average synergy score value of all biological replicates (*n* = 3) was equal to or greater than 10, antagonistic when less than −10, and additive when the value was between −10 and 10 [47].

### 4.15. Statistical Analysis

A comparison between the two groups was calculated using the Mann–Whitney U test. The Kruskal–Wallis test with Dunn’s post-test for multiple comparisons was used to evaluate differences among more than two groups. Differences were considered statistically significant when *p* < 0.05. Statistical analyses were performed by the GraphPad Prism version 8.1.1 for Mac OS X statistics software (GraphPad Software).

## 5. Conclusions

Although the metabolic vulnerabilities associated with different types of tumors have only recently become evident, their impact on the discovery of effective treatments is enormous. Our study underscores the promising potential of PDPDs such as P2Et and Anamu-SC as therapeutic agents for AL. We demonstrated its anti-leukemic activity using several lines of lymphoid and myeloid leukemia that present metabolic differences. Our findings indicate that these PDPDs not only reduce viability and proliferation but also impact mitochondrial function. P2Et induces potentially reversible mitochondrial damage on tumor cells. In contrast, Anamu-SC and Esperanza induce a more substantial irreversible mitochondrial impairment, which is more selective for tumor cells treated with Esperanza, leading to the classification of these PDPDs as mitocans. Moreover, their combination with traditional chemotherapeutics enhances cytostatic effects, suggesting a practical avenue for therapeutic intervention. However, further research is warranted to elucidate the intricate metabolic requirements of leukemia cells, their interactions with PDPDs, and optimal treatment strategies tailored to specific subtypes. Overall, our findings highlight the importance of metabolic alterations for the development of anti-leukemia therapies and pave the way for future research in this field.

## Figures and Tables

**Table 1:** List of altered and common metabolites in the lysates of K562 cells treated with Anamu-SC and Esperanza; *p*-value *: corresponding to the *p* values calculated by the Benjamini–Hochberg false discovery rate post hoc correction (FDR < 0.05); VIP: variable importance in projection; PC: phosphatidylcholines; FC: fold change, 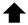 metabolites present only in Anamu-SC extract or Esperanza-treated cell lysates.

Metabolites Altered by *P. alliacea* Extracts							
Compound	Formula	Anamu-SC		*p* Value	Esperanza		*p* Value
		FC	VIP		FC	VIP	
Indoles and derivatives							
Tryptophanol	C_10_H_11_NO	0.45	1.83	0.0021 *	0.61	-	0.009 *
Indoleacrylic acid	C_11_H_9_NO_2_	1.42	1.75	0.0087 *	1.36	1.34	-
Glycerophospholipids							
PC (18:3)	C_26_H_48_NO_7_P	116.2	6.49	0.0022 *	3.37	-	0.009 *
Fatty Acyls							
Propionylcarnitine	C_10_H_19_NO_4_	0.68	1.44	0.0022 *	0.59	-	0.010 *
Hydroxyoctadecatrienoylcarnitine	C_25_H_43_NO_5_	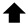	4.52	0.0028 *	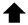	-	0.010 *
Carboxylic acids and derivatives							
Glutathione	C_10_H_17_N_3_O_6_S	0.87	2.71	-	1.31	3.55	0.019 *
Purine nucleosides							
Adenosine monophosphate	C_10_H_14_N_5_O_7_P	3.06	1.79	0.0022 *	0.44	1.05	0.009 *
Organooxygen compounds							
Pantothenate	C_9_H_17_NO_5_	0.72	1.52	-	0.99	1.31	-

## Data Availability

The data presented in this study are available in this article and Supplemental Materials.

## Supplementary Materials

The following supporting information can be downloaded at https://www.mdpi.com/article/10.3390/molecules30081783/s1, Table S1: Summary of clinical data of the patients collected; Table S2: IC_50_ values of metabolic modulators after 48 h of treatment on leukemic cells; Table S3: The proliferation index is presented as the fold change versus the vehicle of each metabolism-modulating treatment and each cell line; Table S4: Metabolites identified in the K562 cell lysates treated with Anamu-SC; Table S5. The ZIP score was obtained from the combination of metabolic modulators and chemotherapeutics on lymphoid and myeloid leukemia cells; Figure S1: Analysis of mitochondrial morphology in K562 myeloid cells treated with P2Et and Anamu-SC; Figure S2: Analysis of mitochondrial morphology in K562 myeloid cells treated with Esperanza; Figure S3: Dose–response viability curve of K562, 3T3, HPdLF, and PBMCs cell treated with P2Et extract; Figure S4: Metabolic differences in K562 cells treated with Anamu-SC vs. vehicle, revealed by untargeted metabolome profiling; Figure S5: Evaluation of the cytotoxic activity of chemotherapeutic drugs on leukemic lines; Figure S6: Assessment of the K562 cells’ viability was conducted by treating them for 24 h with the extracts and then for 12 h with the chemotherapy; Figure S7: A 2D graph prepared by the SynergyFinder^+^ software that shows the synergistic effect between Ara-C and the Esperanza extract on the K562 line.

## References

[1] Swerdlow S.H., Campoo E., Lee N., Jaffe E.S., Pileri S.A., Stein H., Thiele J. (2017). WHO Classification of Tumors for Haematopoietic and Lymphoid Tissues.

[2] Kreitz J., Schönfeld C., Seibert M., Stolp V., Alshamleh I., Oellerich T., Steffen B., Schwalbe H., Schnütgen F., Kurrle N. (2019). Metabolic Plasticity of Acute Myeloid Leukemia. Cells.

[3] Di Martino L., Tosello V., Peroni E., Piovan E. (2021). Insights on Metabolic Reprogramming and Its Therapeutic Potential in Acute Leukemia. Int. J. Mol. Sci..

[4] Loew A., Köhnke T., Rehbeil E., Pietzner A., Weylandt K.-H. (2019). A Role for Lipid Mediators in Acute Myeloid Leukemia. Int. J. Mol. Sci..

[5] Sriskanthadevan S., Jeyaraju D.V., Chung T.E., Prabha S., Xu W., Skrtic M., Jhas B., Hurren R., Gronda M., Wang X. (2015). AML cells have low spare reserve capacity in their respiratory chain that renders them susceptible to oxidative metabolic stress. Blood.

[6] Chaachouay N., Zidane L. (2024). Plant-Derived Natural Products: A Source for Drug Discovery and Development. Drugs Drug Candidates.

[7] Duran M.I., Ballesteros-Ramírez R., Tellez A., Torregrosa L., Olejua P.A., Galvis S., Urueña C., Fiorentino S. (2022). Safety Evaluation in Healthy Colombian Volunteers of P2Et Extract Obtained from *Caesalpinia spinosa*: Design 3+3 Phase I Clinical Trial. Evid.-Based Complement. Altern. Med..

[8] Urueña C., Sandoval T.A., Lasso P., Tawil M., Barreto A., Torregrosa L., Fiorentino S. (2020). Evaluation of chemotherapy and P2Et extract combination in ex-vivo derived tumor mammospheres from breast cancer patients. Sci. Rep..

[9] O’Brien K., Ried K., Binjemain T., Sali A. (2022). Integrative Approaches to the Treatment of Cancer. Cancers.

[10] Wang Y.J., Liao C.C., Chen H.J., Hsieh C.L., Li T.C. (2016). The Effectiveness of Traditional Chinese Medicine in Treating Patients with Leukemia. Evid.-Based Complement. Altern. Med..

[11] Guerra A.R., Duarte M.F., Duarte I.F. (2018). Targeting Tumor Metabolism with Plant-Derived Natural Products: Emerging Trends in Cancer Therapy. J. Agric. Food Chem..

[12] Gomez-Cadena A., Barreto A., Fioretino S., Jandus C. (2020). Immune system activation by natural products and complex fractions: A network pharmacology approach in cancer treatment. Cell Stress.

[13] Sarwar M.S., Zhang H.-J., Tsang S.W. (2018). Perspectives of Plant Natural Products in Inhibition of Cancer Invasion and Metastasis by Regulating Multiple Signaling Pathways. Curr. Med. Chem..

[14] Luz D.A., Pinheiro A.M., Silva M.L., Monteiro M.C., Prediger R.D., Ferraz Maia C.S., Fontes-Júnior E.A. (2016). Ethnobotany, phytochemistry and neuropharmacological effects of *Petiveria alliacea* L. (Phytolaccaceae): A review. J. Ethnopharmacol..

[15] Robledo S.M., Quintero J., Higuita J., Fernández M., Murillo J., Restrepo A., Arbeláez N., Montoya A., Ospina V., Pineda T. (2020). *Caesalpinia spinosa* (Molina) Kuntze: Una nueva promesa para el tratamiento tópico de la leishmaniasis cutánea. Rev. Acad. Colomb. Cienc. Exactas Físicas Nat..

[16] Ma H.Y., Wang C.Q., He H., Yu Z.Y., Tong Y., Liu G., Yang Y.Q., Li L., Pang L., Qi H.Y. (2020). Ethyl acetate extract of *Caesalpinia sappan* L. inhibited acute myeloid leukemia via ROS-mediated apoptosis and differentiation. Phytomedicine.

[17] Tran M.H., Nguyen M.T., Nguyen H.D., Nguyen T.D., Phuong T.T. (2015). Cytotoxic constituents from the seeds of Vietnamese *Caesalpinia sappan*. Pharm. Biol..

[18] Lopes-Martins R.A., Pegoraro D.H., Woisky R., Penna S.C., Sertié J.A. (2002). The anti-inflammatory and analgesic effects of a crude extract of *Petiveria alliacea* L. (Phytolaccaceae). Phytomedicine.

[19] Christie S.L., Levy A. (2013). Evaluation of the Hypoglycaemic Activity of *Petiveria alliacea* (Guinea Hen Weed) Extracts in Normoglycaemic and Diabetic Rat Models. West Indian Med. J..

[20] Gunawan V., Soetjipto H., Mustika A. (2020). Hypoglicemic and Antioxidant Activity of *Petiveria alliacea* in Diabetic Rat Models. Biomol. Health Sci. J..

[21] dos Santos Júnior H.M., Oliveira D.F., de Carvalho D.A., Pinto J.M., Campos V.A., Mourão A.R., Pessoa C., de Moraes M.O., Costa-Lotufo L.V. (2010). Evaluation of native and exotic Brazilian plants for anticancer activity. J. Nat. Med..

[22] Murillo N., Lasso P., Urueña C., Pardo-Rodriguez D., Ballesteros-Ramírez R., Betancourt G., Rojas L., Cala M.P., Fiorentino S. (2023). *Petiveria alliacea* Reduces Tumor Burden and Metastasis and Regulates the Peripheral Immune Response in a Murine Myeloid Leukemia Model. Int. J. Mol. Sci..

[23] Prieto K., Cao Y., Mohamed E., Trillo-Tinoco J., Sierra R.A., Urueña C., Sandoval T.A., Fiorentino S., Rodriguez P.C., Barreto A. (2019). Polyphenol-rich extract induces apoptosis with immunogenic markers in melanoma cells through the ER stress-associated kinase PERK. Cell Death Discov..

[24] Sandoval T.A., Urueña C.P., Llano M., Gómez-Cadena A., Hernández J.F., Sequeda L.G., Loaiza A.E., Barreto A., Li S., Fiorentino S. (2016). Standardized Extract from *Caesalpinia spinosa* is Cytotoxic over Cancer Stem Cells and Enhance Anticancer Activity of Doxorubicin. Am. J. Chin. Med..

[25] Gomez-Cadena A., Uruenã C., Prieto K., Martinez-Usatorre A., Donda A., Barreto A., Romero P., Fiorentino S. (2016). Immune-system-dependent anti-tumor activity of a plant-derived polyphenol rich fraction in a melanoma mouse model. Cell Death Dis..

[26] Lasso P., Gomez-Cadena A., Urueña C., Donda A., Martinez-Usatorre A., Barreto A., Romero P., Fiorentino S. (2018). Prophylactic vs. therapeutic treatment with P2Et polyphenol-rich extract has opposite effects on tumor growth. Front. Oncol..

[27] Arévalo-Ferrin J.J., García-Ortiz J.A., Arevalo-Olaya C.M., Quijano-Gómez S.M., Fiorentino-Gómez S., Rodríguez-Pardo V.M. (2023). Plant-derived extracts P2Et and Anamu-SC affect NO and ROS levels in leukemic cells. Univ. Sci..

[28] Hernández J.F., Urueña C.P., Cifuentes M.C., Sandoval T.A., Pombo L.M., Castañeda D., Asea A., Fiorentino S. (2014). A *Petiveria alliacea* standardized fraction induces breast adenocarcinoma cell death by modulating glycolytic metabolism. J. Ethnopharmacol..

[29] Ballesteros-Ramírez R., Aldana E., Herrera M.V., Urueña C., Rojas L.Y., Echeverri L.F., Costa G.M., Quijano S., Fiorentino S. (2020). Preferential Activity of *Petiveria alliacea* Extract on Primary Myeloid Leukemic Blast. Evid.-Based Complement. Altern. Med..

[30] DeBerardinis R.J., Chandel N.S. (2016). Fundamentals of cancer metabolism. Sci. Adv..

[31] Patra S., Elahi N., Armorer A., Arunachalam S., Omala J., Hamid I., Ashton A.W., Joyce D., Jiao X., Pestell R.G. (2021). Mechanisms Governing Metabolic Heterogeneity in Breast Cancer and Other Tumors. Front. Oncol..

[32] Saengboonmee C., Seubwai W., Pairojkul C., Wongkham S. (2016). High glucose enhances progression of cholangiocarcinoma cells via STAT3 activation. Sci. Rep..

[33] Yucel B., Altundağ Kara S., Cekmen M.B., Ada S., Demircan Tan B. (2022). STAT3 mediated regulation of glucose metabolism in leukemia cells. Gene.

[34] You H., Wang D., Wei L., Chen J., Li H., Liu Y. (2022). Deferoxamine Inhibits Acute Lymphoblastic Leukemia Progression through Repression of ROS/HIF-1α, Wnt/β-Catenin, and p38MAPK/ERK Pathways. J. Oncol..

[35] Dong C., Zhang N.J., Zhang L.J. (2021). Oxidative stress in leukemia and antioxidant treatment. Chin. Med. J..

[36] Mondet J., Lo Presti C., Chevalier S., Bertrand A., Tondeur S., Blanchet S., Mc Leer A., Pernet-Gallay K., Mossuz P. (2021). Mitochondria in human acute myeloid leukemia cell lines have ultrastructural alterations linked to deregulation of their respiratory profiles. Exp. Hematol..

[37] Chen T.R. (1985). Modal karyotype of human leukemia cell line, K562 (ATCC CCL 243). Cancer Genet. Cytogenet..

[38] Baykal-Köse S., Acikgoz E., Yavuz A.S., Gönül Geyik Ö., Ateş H., Sezerman O.U., Özsan G.H., Yüce Z. (2020). Adaptive phenotypic modulations lead to therapy resistance in chronic myeloid leukemia cells. PLoS ONE.

[39] TeSlaa T., Teitell M.A. (2014). Techniques to monitor glycolysis. Methods Enzymol..

[40] Hernández J.F., Urueña C.P., Sandoval T.A., Cifuentes M.C., Formentini L., Cuezva J.M., Fiorentino S. (2017). A cytotoxic *Petiveria alliacea* dry extract induces ATP depletion and decreases β-F1-ATPase expression in breast cancer cells and promotes survival in tumor-bearing mice. Braz. J. Pharmacogn..

[41] Adebayo M., Singh S., Singh A.P., Dasgupta S. (2021). Mitochondrial fusion and fission: The fine-tune balance for cellular homeostasis. FASEB J..

[42] Zorova L.D., Popkov V.A., Plotnikov E.Y., Silachev D.N., Pevzner I.B., Jankauskas S.S., Babenko V.A., Zorov S.D., Balakireva A.V., Juhaszova M. (2018). Mitochondrial membrane potential. Anal. Biochem..

[43] Horn A., Raavicharla S., Shah S., Cox D., Jaiswal J.K. (2020). Mitochondrial fragmentation enables localized signaling required for cell repair. J. Cell Biol..

[44] Rojas L., Pardo-Rodriguez D., Urueña C., Lasso P., Arévalo C., Cala M.P., Fiorentino S. (2023). Effect of *Petiveria alliacea* Extracts on Metabolism of K562 Myeloid Leukemia Cells. Int. J. Mol. Sci..

[45] Fasinu P.S., Rapp G.K. (2019). Herbal Interaction with Chemotherapeutic Drugs-A Focus on Clinically Significant Findings. Front. Oncol..

[46] Le Gal K., Ibrahim M.X., Wiel C., Sayin V.I., Akula M.K., Karlsson C., Dalin M.G., Akyürek L.M., Lindahl P., Nilsson J. (2015). Antioxidants can increase melanoma metastasis in mice. Sci. Transl. Med..

[47] Ianevski A., Giri A.K., Aittokallio T. (2022). SynergyFinder 3.0: An interactive analysis and consensus interpretation of multi-drug synergies across multiple samples. Nucleic Acids Res..

[48] Egan G., Schimmer A.D. (2023). Contribution of metabolic abnormalities to acute myeloid leukemia pathogenesis. Trends Cell Biol..

[49] Chen J., Huang C., Zhu Y., Dong L., Cao W., Sun L., Sun H., Wan D., Liu Y., Zhang Z. (2015). Identification of similarities and differences between myeloid and lymphoid acute leukemias using a gene-gene interaction network. Pathol.-Res. Pract..

[50] Prieto-Bermejo R., Romo-González M., Pérez-Fernández A., Ijurko C., Hernández-Hernández Á. (2018). Reactive oxygen species in haematopoiesis: Leukaemic cells take a walk on the wild side. J. Exp. Clin. Cancer Res..

[51] Nielsen I., Groth-Pedersen L., Dicroce-Giacobini J., Jonassen A.S.H., Mortensen M., Bilgin M., Schmiegelow K., Jäättelä M., Maeda K. (2020). Cationic amphiphilic drugs induce elevation in lysoglycerophospholipid levels and cell death in leukemia cells. Metabolomics.

[52] Musharraf S.G., Siddiqui A.J., Shamsi T., Naz A. (2017). SERUM metabolomics of acute lymphoblastic leukaemia and acute myeloid leukaemia for probing biomarker molecules. Hematol. Oncol..

[53] Morad H.M., Abou-Elzahab M.M., Aref S., El-Sokkary A.M.A. (2022). Diagnostic Value of ^1^H NMR-Based Metabolomics in Acute Lymphoblastic Leukemia, Acute Myeloid Leukemia, and Breast Cancer. ACS Omega.

[54] Hao X., Hui-Tao Z., Hong-Wen X., Chun-Lan H., Mei-Zhou H. (2022). Serum Metabolomics Coupling with Clinical Laboratory Indicators Reveal Taxonomic Features of Leukemia. Front. Pharmacol..

[55] Olivas-Aguirre M., Torres-López L., Pottosin I., Dobrovinskaya O. (2020). Phenolic Compounds Cannabidiol, Curcumin and Quercetin Cause Mitochondrial Dysfunction and Suppress Acute Lymphoblastic Leukemia Cells. Int. J. Mol. Sci..

[56] Banella C., Catalano G., Travaglini S., Pelosi E., Ottone T., Zaza A., Guerrera G., Angelini D.F., Niscola P., Divona M. (2022). Ascorbate Plus Buformin in AML: A Metabolic Targeted Treatment. Cancers.

[57] Hasanpourghadi M., Yeng Looi C., Kumar Pandurangan A., Sethi G., Fen Wong W., Rais Mustafa M. (2017). Phytometabolites Targeting the Warburg Effect in Cancer Cells: A Mechanistic Review. Curr. Drug Targets.

[58] Hlozkova K., Pecinova A., Alquezar-Artieda N., Pajuelo-Reguera D., Simcikova M., Hovorkova L., Rejlova K., Zaliova M., Mracek T., Kolenova A. (2020). Metabolic profile of leukemia cells influences treatment efficacy of L-asparaginase. BMC Cancer.

[59] Nishiura T., Suzuki K., Kawaguchi T., Nakao H., Kawamura N., Taniguchi M., Kanayama Y., Yonezawa T., Iizuka S., Taniguchi N. (1992). Elevated serum manganese superoxide dismutase in acute leukemias. Cancer Lett..

[60] Paydas S., Yuregir G.T., Sahin B., Seyrek E., Burgut R. (1995). Intracellular glutathione content in leukemias. Oncology.

[61] Montaño A., Forero-Castro M., Marchena-Mendoza D., Benito R., Hernández-Rivas J. (2018). New Challenges in Targeting Signaling Pathways in Acute Lymphoblastic Leukemia by NGS Approaches: An Update. Cancers.

[62] Rodrigues A., Costa R.G.A., Silva S.L.R., Dias I., Dias R.B., Bezerra D.P. (2021). Cell signaling pathways as molecular targets to eliminate AML stem cells. Crit. Rev. Oncol./Hematol..

[63] Puente-Moncada N., Turos-Cabal M., Sánchez A.M., Antolín I., Herrera F., Rodriguez J., Duarte C., Rodriguez C., Martín V. (2020). Role of glucose metabolism in the differential antileukemic effect of melatonin on wild-type and FLT3-ITD mutant cells. Oncol. Rep..

[64] Lucas D.M., Still P.C., Bueno Perez L., Grever M.R., Douglas Kinghorn A. (2010). Potential of Plant-Derived Natural Products in the Treatment of Leukemia and Lymphoma. Curr. Drug Targets.

[65] Maher T., Ahmad Raus R., Daddiouaissa D., Ahmad F., Adzhar N.S., Latif E.S., Abdulhafiz F., Mohammed A. (2021). Medicinal Plants with Anti-Leukemic Effects: A Review. Molecules.

[66] Lapa B., Gonçalves A.C., Jorge J., Alves R., Pires A.S., Abrantes A.M., Coucelo M., Abrunhosa A., Botelho M.F., Nascimento-Costa J.M. (2020). Acute myeloid leukemia sensitivity to metabolic inhibitors: Glycolysis showed to be a better therapeutic target. Med. Oncol..

[67] Panina S.B., Baran N., Brasil da Costa F.H., Konopleva M., Kirienko N.V. (2019). A mechanism for increased sensitivity of acute myeloid leukemia to mitotoxic drugs. Cell Death Dis..

[68] Panina S.B., Pei J., Baran N., Konopleva M., Kirienko N.V. (2020). Utilizing Synergistic Potential of Mitochondria-Targeting Drugs for Leukemia Therapy. Front. Oncol..

[69] De Beauchamp L., Himonas E., Helgason G.V. (2022). Mitochondrial metabolism as a potential therapeutic target in myeloid leukaemia. Leukemia.

[70] Pumiputavon K., Chaowasku T., Saenjum C., Osathanunkul M., Wungsintaweekul B., Chawansuntati K., Lithanatudom P., Wipasa J. (2019). Cytotoxic and cytostatic effects of four Annonaceae plants on human cancer cell lines. In Vitr. Cell. Dev. Biol.-Anim..

[71] Comin-Anduix B., Boros L.G., Marin S., Boren J., Callol-Massot C., Centelles J.J., Torres J.L., Agell N., Bassilian S., Cascante M. (2002). Fermented Wheat Germ Extract Inhibits Glycolysis/Pentose Cycle Enzymes and Induces Apoptosis through Poly(ADP-ribose) Polymerase Activation in Jurkat T-cell Leukemia Tumor Cells. J. Biol. Chem..

[72] Lee E.A., Angka L., Rota S.G., Hanlon T., Mitchell A., Hurren R., Wang X.M., Gronda M., Boyaci E., Bojko B. (2015). Targeting mitochondria with avocatin B induces selective leukemia cell death. Cancer Res..

[73] Mahbub A., Maitre C., Haywood-Small S., Mcdougall G., Cross N., Jordan-Mahy N. (2013). Differential Effects of Polyphenols on Proliferation and Apoptosis in Human Myeloid and Lymphoid Leukemia Cell Lines. Anti-Cancer Agents Med. Chem..

[74] Ashrafizadeh M., Zarrabi A., Mirzaei S., Hashemi F., Samarghandian S., Zabolian A., Hushmandi K., Ang H.L., Sethi G., Kumar A.P. (2021). Gallic acid for cancer therapy: Molecular mechanisms and boosting efficacy by nanoscopical delivery. Food Chem. Toxicol..

[75] Gu R., Zhang M., Meng H., Xu D., Xie Y. (2018). Gallic acid targets acute myeloid leukemia via Akt/mTOR-dependent mitochondrial respiration inhibition. Biomed. Pharmacother..

[76] Mamouni K., Kallifatidis G., Lokeshwar B.L. (2021). Targeting Mitochondrial Metabolism in Prostate Cancer with Triterpenoids. Int. J. Mol. Sci..

[77] Stevens J.F., Revel J.S., Maier C.S. (2018). Mitochondria-Centric Review of Polyphenol Bioactivity in Cancer Models. Antioxid. Redox Signal..

[78] Yang J.H., Chou C.C., Cheng Y.W., Sheen L.Y., Chou M.C., Yu H.S., Wei Y.H., Chung J.G. (2004). Effects of glycolic acid on the induction of apoptosis via caspase-3 activation in human leukemia cell line (HL-60). Food Chem. Toxicol..

[79] Samimi S., Ardestani M.S., Dorkoosh F.A. (2021). Preparation of carbon quantum dots- quinic acid for drug delivery of gemcitabine to breast cancer cells. J. Drug Deliv. Sci. Technol..

[80] Mookerjee S.A., Goncalves R.L.S., Gerencser A.A., Nicholls D.G., Brand M.D. (2015). The contributions of respiration and glycolysis to extracellular acid production. Biochim. Biophys. Acta.

[81] van der Veen J.N., Kennelly J.P., Wan S., Vance J.E., Vance D.E., Jacobs R.L. (2017). The critical role of phosphatidylcholine and phosphatidylethanolamine metabolism in health and disease. Biochim. Biophys. Acta Biomembr..

[82] Dong J., Ye F., Lin J., He H., Song Z. (2023). The metabolism and function of phospholipids in Mitochondria. Mitochondrial Commun..

[83] McCann M.R., George De la Rosa M.V., Rosania G.R., Stringer K.A. (2021). L-Carnitine and Acylcarnitines: Mitochondrial Biomarkers for Precision Medicine. Metabolites.

[84] Sharma S., Black S.M. (2009). Carnitine Homeostasis, Mitochondrial Function, and Cardiovascular Disease. Drug Discov. Today Dis. Mech..

[85] Soler-Agesta R., Anel A., Galluzzi L. (2023). Mitochondrial control of antigen presentation in cancer cells. Cancer Cell.

[86] Chen Y., Liang Y., Luo X., Hu Q. (2020). Oxidative resistance of leukemic stem cells and oxidative damage to hematopoietic stem cells under pro-oxidative therapy. Cell Death Dis..

[87] Gorini S., De Angelis A., Berrino L., Malara N., Rosano G., Ferraro E. (2018). Chemotherapeutic drugs and mitochondrial dysfunction: Focus on doxorubicin, trastuzumab, and sunitinib. Oxidative Med. Cell. Longev..

[88] Urueña C., Cifuentes C., Castañeda D., Arango A., Kaur P., Asea A., Fiorentino S. (2008). *Petiveria alliacea* extracts uses multiple mechanisms to inhibit growth of human and mouse tumoral cells. BMC Complement. Altern. Med..

[89] Padilla-Arellanes S., Salgado-Garciglia R., Báez-Magaña M., Ochoa-Zarzosa A., López-Meza J.E. (2021). Cytotoxicity of a Lipid-Rich Extract from Native Mexican Avocado Seed (*Persea americana* var. drymifolia) on Canine Osteosarcoma D-17 Cells and Synergistic Activity with Cytostatic Drugs. Molecules.

[90] Mahbub A., Le Maitre C., Haywood-Small S., Cross N., Jordan-Mahy N. (2015). Polyphenols act synergistically with doxorubicin and etoposide in leukaemia cell lines. Cell Death Discov..

[91] Yao S., Zhong L., Chen M., Zhao Y., Li L., Liu L., Xu T., Xiao C., Gan L., Shan Z. (2021). Epigallocatechin-3-gallate promotes all-trans retinoic acid-induced maturation of acute promyelocytic leukemia cells via PTEN. Int. J. Oncol..

[92] Dakik H., El Dor M., Bourgeais J., Kouzi F., Herault O., Gouilleux F., Zibara K., Mazurier F. (2022). Diphenyleneiodonium Triggers Cell Death of Acute Myeloid Leukemia Cells by Blocking the Mitochondrial Respiratory Chain, and Synergizes with Cytarabine. Cancers.

[93] Ballesteros-Ramírez R., Lasso P., Urueña C., Saturno J., Fiorentino S. (2024). Assessment of Acute and Chronic Toxicity in Wistar Rats (*Rattus norvegicus*) and New Zealand Rabbits (*Oryctolagus cuniculus*) of an Enriched Polyphenol Extract Obtained from *Caesalpinia spinosa*. J. Toxicol..

[94] Lasso P., Rojas L., Arévalo C., Urueña C., Murillo N., Barreto A., Costa G.M., Fiorentino S. (2022). *Tillandsia usneoides* Extract Decreases the Primary Tumor in a Murine Breast Cancer Model but Not in Melanoma. Cancers.

[95] Soares T., Rodrigues D., Sarraguça M., Rocha S., Lima J.L.F.C., Ribeiro D., Fernandes E., Freitas M. (2019). Optimization of experimental settings for the assessment of reactive oxygen species production by human blood. Oxidative Med. Cell. Longev..

[96] Mao Y., Hoffman T., Wu A., Kohn J. (2018). An Innovative Laboratory Procedure to Expand Chondrocytes with Reduced Dedifferentiation. Cartilage.

[97] Carlosama C., Arévalo C., Jimenez M.C., Lasso P., Urueña C., Fiorentino S., Barreto A. (2024). Triple negative breast cancer migration is modified by mitochondrial metabolism alteration induced by natural extracts of *C. spinosa* and *P. alliacea*. Sci. Rep..

[98] Hamon M.P., Gergondey R., L’Honoré A., Friguet B. (2020). Mitochondrial Lon protease-depleted HeLa cells exhibit proteome modifications related to protein quality control, stress response and energy metabolism. Free Radic. Biol. Med..

[99] Koopman W.J.H., Visch H.J., Smeitink J.A.M., Willems P.H.G.M. (2006). Simultaneous quantitative measurement and automated analysis of mitochondrial morphology, mass, potential, and motility in living human skin fibroblasts. Cytom. Part A J. Int. Soc. Anal. Cytol..

[100] Tronstad K., Nooteboom M., Nilsson L., Nikolaisen J., Sokolewicz M., Grefte S., Pettersen I., Dyrstad S., Hoel F., Willems P. (2014). Regulation and Quantification of Cellular Mitochondrial Morphology and Content. Curr. Pharm. Des..

[101] Hodneland Nilsson L.I., Nitschke Pettersen I.K., Nikolaisen J., Micklem D., Avsnes Dale H., Vatne Røsland G., Lorens J., Tronstad K.J. (2015). A new live-cell reporter strategy to simultaneously monitor mitochondrial biogenesis and morphology. Sci. Rep..

[102] Castañeda D.M., Pombo L.M., Urueña C.P., Hernandez J.F., Fiorentino S. (2012). A gallotannin-rich fraction from *Caesalpinia spinosa* (Molina) Kuntze displays cytotoxic activity and raises sensitivity to doxorubicin in a leukemia cell line. BMC Complement. Altern. Med..

[103] Blaženović I., Kind T., Ji J., Fiehn O. (2018). Software Tools and Approaches for Compound Identification of LC-MS/MS Data in Metabolomics. Metabolites.

