# Supplementary material for: Modulation of Tumor Metabolism in Acute Leukemia by Plant-Derived Polymolecular Drugs and Their Effects on Mitochondrial Function
Source: Molecules. 2025 Apr 16;30(8):1783. doi: 10.3390/molecules30081783 (PMC12029889; doi:10.3390/molecules30081783)
Supplement: Supplementary file 1 [file molecules-30-01783-s001.zip › molecules-3524805-supplementary.pdf]

Supplemental material

Tables

**Number**	**Consecutive**	**Diagnosis**	**Gender**	**Age**	**Genetic alteration**	**Risk**	**Basal MFI H_2DCFDA**
1	61885-9	B-ALL	F	65	t(9;22)	High	Not evaluated
2	61885-17	B-ALL	F	47	t(9;22)	High	Not evaluated
3	61885-46	B-ALL	F	57	t(9;22)	High	1,921
4	61885-48	B-ALL	M	35	None	Low	1,804.5
5	61885-58	B-ALL	M	43	None	High	1,33
6	61885-60	B-ALL	M	57	t(9;22)	High	1,021.5
7	61885-74	B-ALL	F	21	None	Intermediate	548
8	61885-79	B-ALL	M	39	t(9;22)	High	242
9	61885-80	B-ALL	F	23	None	Intermediate	344.5
10	61885-6	AML	M	67	None	Intermediate	Not evaluated
11	61885-23	AML	F	49	FLT3+, NMP1+	Intermediate	Not evaluated
12	61885-49	AML	M	73	Complex karyotype	High	16,393.5
13	61885-50	AML	M	76	None	High	11,028.5
14	61885-57	AML	F	67	Complex karyotype	High	373
15	61885-64	AML	F	52	Unknown	Intermediate	3,135
16	61885-69	AML	M	45	None	Intermediate	1,215.5
17	61885-78	AML	M	59	Inv(16)	Low	144.5

Table S1. Summary of clinical data of the patients collected. B-ALL: B-acute lymphoid leukemia, AML: acute myeloid leukemia, F: Female, M: Male, FLT3: FMS-like tyrosine kinase 3, NMP1: nucleophosmin 1.

Cytotoxicity 48 h	AA [mM]		P2Et [μg/mL]		2-DG [μM]		Anamu-SC [μg/mL]	
	IC_{50}	DS	IC_{50}	DS	IC_{50}	DS	IC_{50}	DS
**Molt-4**	0.48	± 0.08	215.1	± 19.9	65.2	± 9.5	189.8	± 13.1
**Jurkat**	>50	N/A	152.1	± 30.9	255.2	± 35.7	105.8	± 25.8
**Reh**	>50	N/A	253.2	± 22.7	164.3	± 32.3	53.1	± 30.9
**K562**	>50	N/A	178.1	± 27.1	1,384	± 412	294	± 130.2
**OCI-AML3**	>50	N/A	194.5	± 47.3	1,757	± 420.8	130.2	± 26.1
**U937**	5.8	± 1.15	41.2	± 3.5	69.6	± 10.6	66.2	± 8.9

More sensitive

Less sensitive

Table S2. IC_{50} values of metabolic modulators after 48 h of treatment on leukemic cells. The MTT-calculated IC_{50} ± SD values for AA, P2Et, 2-DG, and Anamu-SC are presented. The data are shown as the mean ± SD of three different experiments executed in triplicate. AA: ascorbic acid, 2-DG: 2-Desoxy-D-glucose.

AA [mM] IC50 or standar concentration	Molt-4				Jurkat				Reh				K562				OCI-AML3				U937			
	Vehicle		5 mM		Vehicle		5 mM		Vehicle		5 mM		Vehicle		5 mM		Vehicle		5 mM		Vehicle		IC50	
	Index	DS	Index	DS	Index	DS	Index	DS	Index	DS	Index	DS	Index	DS	Index	DS	Index	DS	Index	DS	Index	DS	Index	DS
12 h	0,76	0,24	0,201	0,089	0,375	0,158	0,000	0,000	0,32	0,08	-0,419	0,137	1,20	0,09	1,038	0,050	0,13	0,19	0,259	0,171	0,72	0,06	0,536	0,071
24 h	2,57	0,03	-1,164	0,228	1,203	0,089	0,633	0,066	1,44	0,08	0,405	0,201	2,22	0,07	1,869	0,140	1,56	0,17	1,139	0,093	2,12	0,12	0,995	0,205
48 h	4,79	0,11	-2,034	0,415	1,635	0,066	0,841	0,228	2,42	0,13	1,038	0,050	3,76	0,08	3,060	0,074	2,41	0,12	2,191	0,022	3,54	0,07	3,254	0,108
72 h	5,17	0,19	-3,831	0,709	2,340	0,101	0,962	0,158	3,05	0,01	2,274	0,233	4,08	0,05	3,581	0,069	3,76	0,23	3,994	0,110	4,91	0,04	4,793	0,037
P2Et [μg/mL]	Molt-4				Jurkat				Reh				K562				OCI-AML3				U937			
	Vehicle		IC50		Vehicle		IC50		Vehicle		IC50		Vehicle		IC50		Vehicle		IC50		Vehicle		IC50	
	Index	DS	Index	DS	Index	DS	Index	DS	Index	DS	Index	DS	Index	DS	Index	DS	Index	DS	Index	DS	Index	DS	Index	DS
12 h	0,76	0,24	0,312	0,247	0,38	0,16	0,000	0,000	0,32	0,08	0,201	0,089	1,20	0,09	0,995	0,205	0,13	0,19	0,069	0,097	0,72	0,06	0,069	0,097
24 h	2,57	0,03	-0,531	0,294	1,20	0,09	0,433	0,076	1,44	0,08	0,483	0,146	2,22	0,07	1,001	0,102	1,56	0,17	0,483	0,146	2,12	0,12	-0,419	0,137
48 h	4,79	0,11	-0,871	0,186	1,64	0,07	0,322	0,082	2,42	0,13	0,243	0,344	3,76	0,08	0,536	0,071	2,41	0,12	-0,161	0,228	3,54	0,07	-0,238	0,120
72 h	5,17	0,19	-2,829	0,709	2,34	0,10	-0,238	0,120	3,05	0,01	-0,531	0,294	4,08	0,05	1,103	0,143	3,76	0,23	-2,829	0,709	4,91	0,04	-3,330	0,000
2-DG [μM]	Molt-4				Jurkat				Reh				K562				OCI-AML3				U937			
	Vehicle		IC50		Vehicle		IC50		Vehicle		IC50		Vehicle		IC50		Vehicle		IC50		Vehicle		IC50	
	Index	DS	Index	DS	Index	DS	Index	DS	Index	DS	Index	DS	Index	DS	Index	DS	Index	DS	Index	DS	Index	DS	Index	DS
12 h	0,76	0,24	0,889	0,055	0,38	0,16	0,201	0,089	0,32	0,08	0,069	0,097	1,20	0,09	0,954	0,264	0,13	0,19	0,069	0,097	0,72	0,06	0,425	0,228
24 h	2,57	0,03	2,137	0,186	1,20	0,09	0,926	0,108	1,44	0,08	0,573	0,274	2,22	0,07	2,190	0,067	1,56	0,17	0,809	0,058	2,12	0,12	1,910	0,082
48 h	4,79	0,11	4,079	0,097	1,64	0,07	1,324	0,082	2,42	0,13	1,633	0,132	3,76	0,08	3,060	0,074	2,41	0,12	2,158	0,069	3,54	0,07	2,900	0,151
72 h	5,17	0,19	4,828	0,102	2,34	0,10	2,142	0,046	3,05	0,01	3,145	0,023	4,08	0,05	3,726	0,054	3,76	0,23	3,763	0,076	4,91	0,04	4,717	0,055
SC [μg/mL]	Molt-4				Jurkat				Reh				K562				OCI-AML3				U937			
	Vehicle		IC50		Vehicle		IC50		Vehicle		IC50		Vehicle		IC50		Vehicle		IC50		Vehicle		IC50	
12 h	0,76	0,24	0,962	0,158	0,375	0,158	0,138	0,000	0,322	0,082	0,069	0,097	1,20	0,09	0,583	0,137	0,13	0,19	0,000	0,000	0,72	0,06	0,069	0,097
24 h	2,57	0,03	-0,161	0,228	1,203	0,089	0,433	0,076	1,435	0,076	0,885	0,166	2,22	0,07	0,889	0,055	1,56	0,17	-0,419	0,137	2,12	0,12	-0,334	0,257
48 h	4,79	0,11	-1,032	0,415	1,635	0,066	0,069	0,097	2,422	0,134	1,034	0,150	3,76	0,08	0,453	0,445	2,41	0,12	0,375	0,158	3,54	0,07	-1,533	0,294
72 h	5,17	0,19	-2,034	0,415	2,340	0,101	-0,419	0,137	3,052	0,012	0,633	0,066	4,08	0,05	0,259	0,171	3,76	0,23	-1,826	0,709	4,91	0,04	-2,829	0,709

Table S3. The proliferation index is presented as the fold change versus the vehicle of each metabolism modulating treatment and each cell line. IC_{50} or standard concentrations of each treatment were used and cell counts were performed at 12 h, 24 h, 48 h and 72 h. Values from two independent experiments ± SD are shown.

Compound	Formula	Mass	^{a}Rt (min)	Mass Error (ppm)	Adduct	^{b}CV for QC (%)	^{c}FC	^dp value	^{e}VIP	^{f}ID Level
Indoles and derivatives										
Tryptophanol	$C_{10}H_{11}NO$	161.0841	23.34	5	$[M+Na]^+$	4.67	0.45	0.0021*	1.83	3
Indoleacrylic acid	$C_{11}H_9NO_2$	187.0633	8.91	1	$[M+H]^+$	7.93	1.42	0.0087*	1.75	3
Glycerophospholipids										
PC (18:3)	$C_{26}H_{48}NO_7P$	517.3168	22.13	1	$[M+H]^+$	9.50	116.21	0.0022*	6.49	3
Glycerylphosphorylcholine	$C_8H_{20}NO_6P$	257.1028	1.45	2	$[M+H]^+$ /$[M+Na]^+$	5.84	0.55	0.0022*	-	2
PI P-18:0	$C_{27}H_{53}O_{11}P$	584.3326	15.60	2	$[M+H]^+$	3.49	0.87	0.0043*	-	3
Nucleosides and nucleotides										
Hydroxyguanosine	$C_{10}H_{13}N_5O_6$	299.0866	2.20	4	$[M+Na]^+$	12.01	0.72	0.0022*	-	3
Fatty Acyls										
Propionylcarnitine	$C_{10}H_{19}NO_4$	217.1314	2.90	1	$[M+H]^+$	5.11	0.68	0.0022*	1.44	3
Hydroxylinoleoylcarnitine	$C_{25}H_{45}NO_5$	439.3298	21.95	0	$[M+H]^+$	10.97	⬆	0.0028*	1.56	3
Hydroxyoctadecatrienoylcarnitine	$C_{25}H_{43}NO_5$	437.3141	21.31	1	$[M+H]^+$	19.55	⬆	0.0028*	4.52	3
Pentanamide	$C_5H_{11}NO$	101.0841	15.61	6	$[M+H]^+$	6.66	0.73	0.0022*	-	3
Carboxylic acids and derivatives										
Cysteinylglycine	$C_5H_{10}N_2O_3S$	178.0412	2.12	3	$[M+H]^+$	5.08	0.87	-	1.30	2
Glutathione	$C_{10}H_{17}N_3O_6S$	307.0838	2.12	1	$[M+H]^+$	5.72	0.87	-	2.71	2
Purine nucleosides										
Adenosine	$C_{10}H_{13}N_5O_4$	267.0968	2.22	1	$[M+H]^+$	8.59	3.90	0.0043*	1.32	2
Adenosine phosphate disodium	$C_{10}H_{14}N_5O_7P.2Na$	391.0270	1.56	1	$[M+H]^+$	5.32	3.02	0.0022*	1.60	3
Adenosine monophosphate	$C_{10}H_{14}N_5O_7P$	347.0631	1.57	0	$[M+Na]^+$	4.02	3.06	0.0022*	1.79	2
Organooxygen compounds										
Pantothenate	$C_9H_{17}NO_5$	219.1107	4.90	2	$[M+H-H_2O]^-$/$[M+H]^+$	10.07	0.72	-	1.52	3
Organoheterocyclic compounds										
Biotin	$C_{10}H_{16}N_2O_3S$	244.0882	16.04	1	$[M+H]^+$	8.78	1.53	0.0043*	-	3

Table S4. Metabolites identified in the K562 cell lysates treated with Anamu-SC. ^{a}Rt: retention time; ^{b}CV: coefficient of variation in the metabolites in the QC samples; ^{c}FC: Fold change; ^dp value*: corresponding to the *p values* calculated by the Benjamini-Hochberg false discovery rate post hoc correction (FDR < 0.05); ^{e}VIP: variable importance in projection; ^{f}ID level: Identification level. PC: phosphatidylcholines; PI: phosphatidylinositol.
⬆ : metabolites present only in Anamu-SC extract-treated cell lysates.

Lymphoid cells	Molt-4						Jurkat						Reh					
Metabolic modulators + Chemotherapeutics	DOX		MTX		VIN		DOX		MTX		VIN		DOX		MTX		VIN	
	Mean ZIP Score	DS	Mean ZIP Score	DS	Mean ZIP Score	DS	Mean ZIP Score	DS	Mean ZIP Score	DS	Mean ZIP Score	DS	Mean ZIP Score	DS	Mean ZIP Score	DS	Mean ZIP Score	DS
**AA**	1,99	± 3,9	-0,45	± 8,2	-0,19	± 3,3	4,87	± 6,76	-3,03	± 5,43	0,4	± 1,6	0,47	± 5,2	-5,4	± 6,3	-10,04	± 3,2
**P2Et**	-19,8	± 5,8	-11,3	± 3,6	-8,2	± 1,3	-12,74	± 5,57	1,76	± 4,74	-3,8	± 1,0	-16,12	± 3,8	-5,3	± 4,4	-4,67	± 3,6
**2-DG**	-5,5	± 0,9	3,6	± 7,2	-6,1	± 4,5	-3,48	± 8,61	1,14	± 10,74	-4,12	± 2,9	-0,52	± 5,5	1,13	± 6,6	-5,61	± 4,47
**Anamu-SC**	-2,9	± 2,6	0,36	± 3,4	4,3	± 4,8	-7,89	± 5,35	0,37	± 3,38	6,25	± 4,65	-3,09	± 2,0	-0,14	± 4,7	-9,19	± 2,4

Myeloid cells	K562				OCI-AML3				U937			
Metabolic modulators + Chemotherapeutics	IDA		Ara-C		IDA		Ara-C		IDA		Ara-C	
	Mean ZIP Score	DS	Mean ZIP Score	DS	Mean ZIP Score	DS	Mean ZIP Score	DS	Mean ZIP Score	DS	Mean ZIP Score	DS
**AA**	5,3	± 5,7	0,17	± 5,5	4,98	± 8,89	7,26	± 4,07	7,17	± 1,75	0,55	± 7,23
**P2Et**	-6,87	± 2,3	9	± 2,7	-7,9	± 2,92	-2,13	± 6,86	-3,89	± 6,75	-1,9	± 0,41
**2-DG**	-5,74	± 3,7	2,8	± 5	-1,08	± 2,48	-4,93	± 3,46	11,7	± 4,36	3,81	± 5,54
**Anamu-SC**	-12,4	± 0,8	11,84	± 2,3	-10,3	± 3,46	0,98	± 4,7	-8,17	± 7,49	-1,57	± 2,56

Table S5. The ZIP score was obtained from the combination of metabolic modulators and chemotherapeutics on lymphoid and myeloid leukemia cells.

Figures

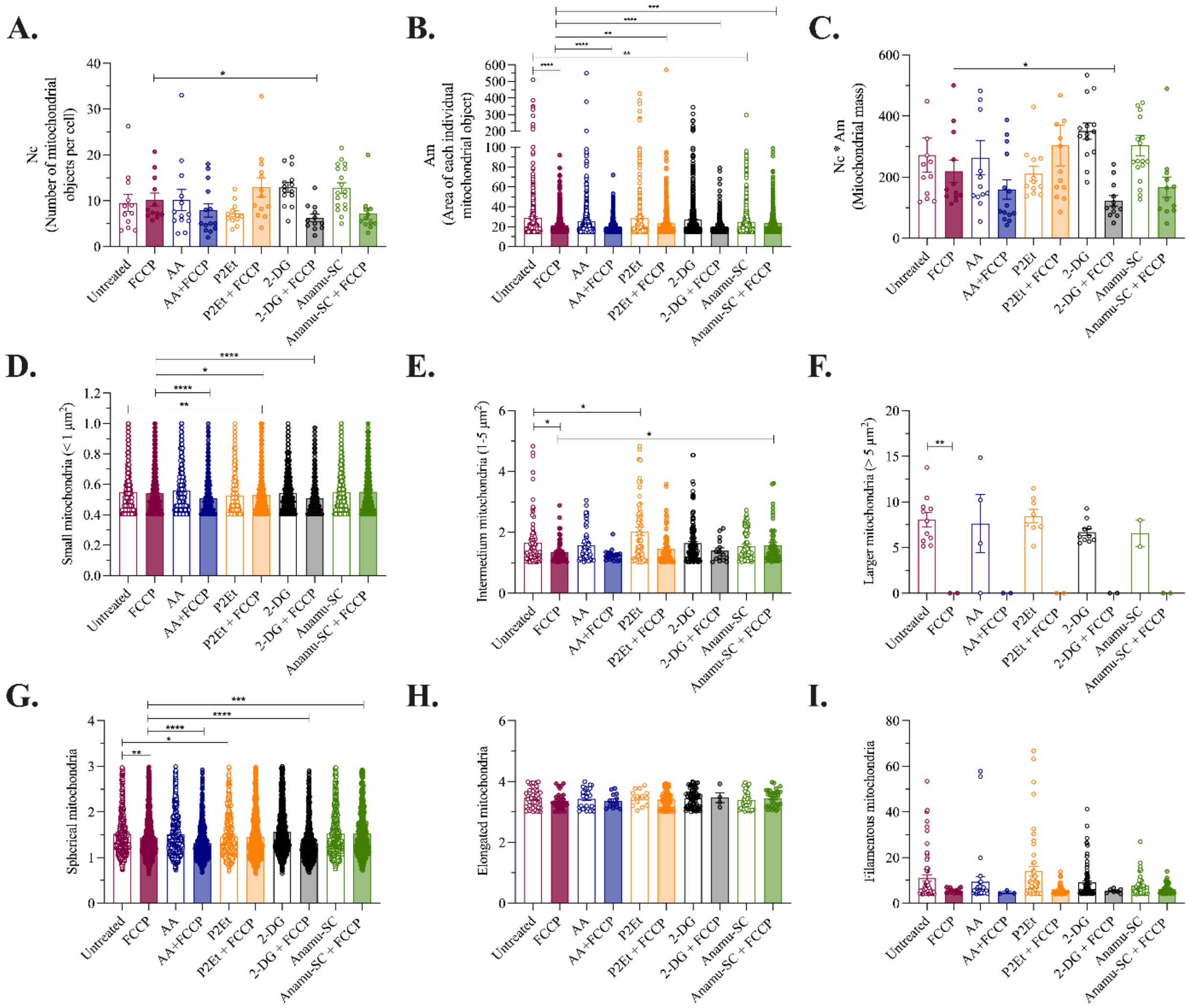

Figure S1. Analysis of mitochondrial morphology in K562 myeloid cells treated with P2Et and Anamu-SC. Comparison between untreated and post-treatment cells with metabolic modulators as single or combined treatments with FCCP 3.5 µM in K562 cells. **A.** Nc. **B.** Am. **C.** Nc * Am. **D.** Small mitochondrial (< 1 µm^2). **E.** Intermedium mitochondrial (1-5 µm^2). **F.** Larger mitochondrial (> 5 µm^2). **G.** Spherical mitochondrial. **H.** Elongated mitochondrial. **I.** Filamentous mitochondrial. Data from two independent experiments and about 80 cells analyzed ± SD are shown.* $p < 0.05$; ** $p < 0.01$; ***$p < 0.001$; ****$p < 0.0001$.

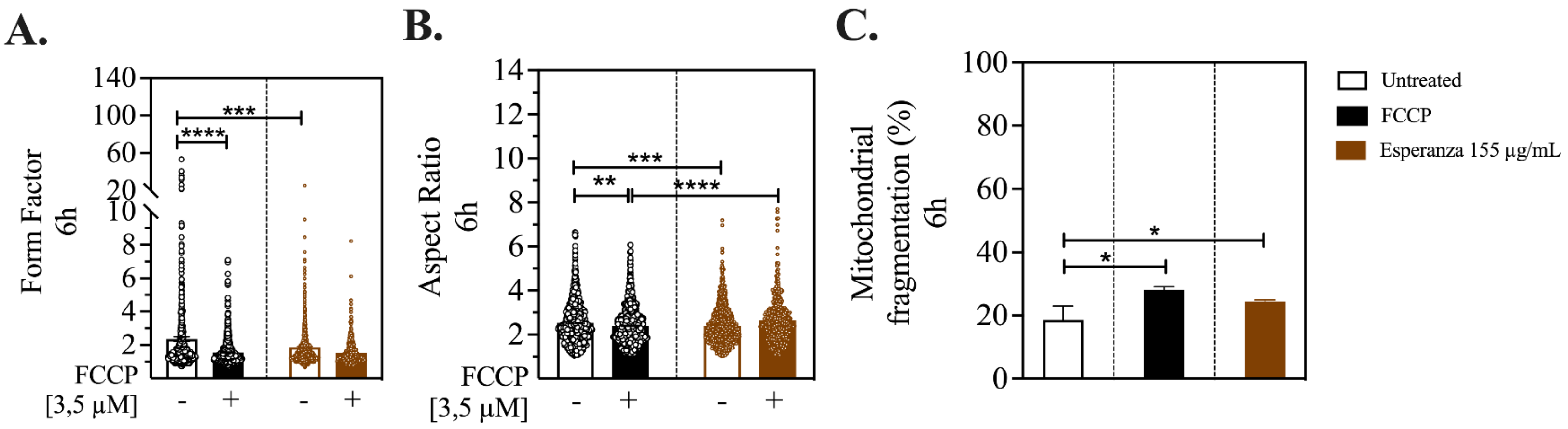

Figure S2. Analysis of mitochondrial morphology in K562 myeloid cells treated with Esperanza. Comparison between untreated and post-treatment cells with Esperanza as single or combined treatments with FCCP 3.5 µM in K562 cells. **A.** Form factor **B.** Aspect ratio. **C.** Mitochondrial fragmentation. Data from two independent experiments and about 80 cells analyzed ± SD are shown.* $p < 0.05$; ** $p < 0.01$; ***p < 0.001; ****$p < 0.0001$.

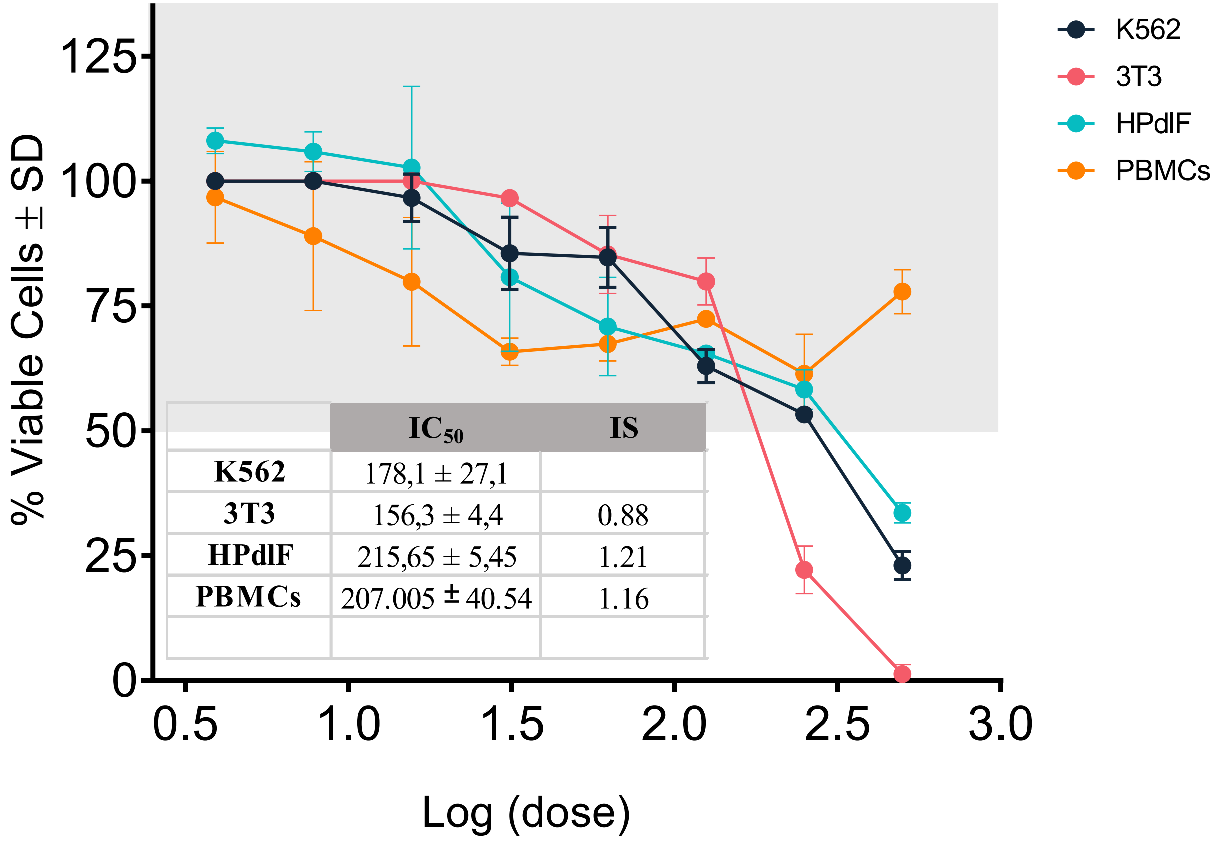

	IC_{50}	IS
**K562**	178,1 ± 27,1	
**3T3**	156,3 ± 4,4	0.88
**HPdlF**	215,65 ± 5,45	1.21
**PBMCs**	207.005 ± 40.54	1.16

Figure S3. Dose-response viability curve of K562, 3T3, HPdLF, and PBMCs cell treated with P2Et extract. IS: Selectivity index

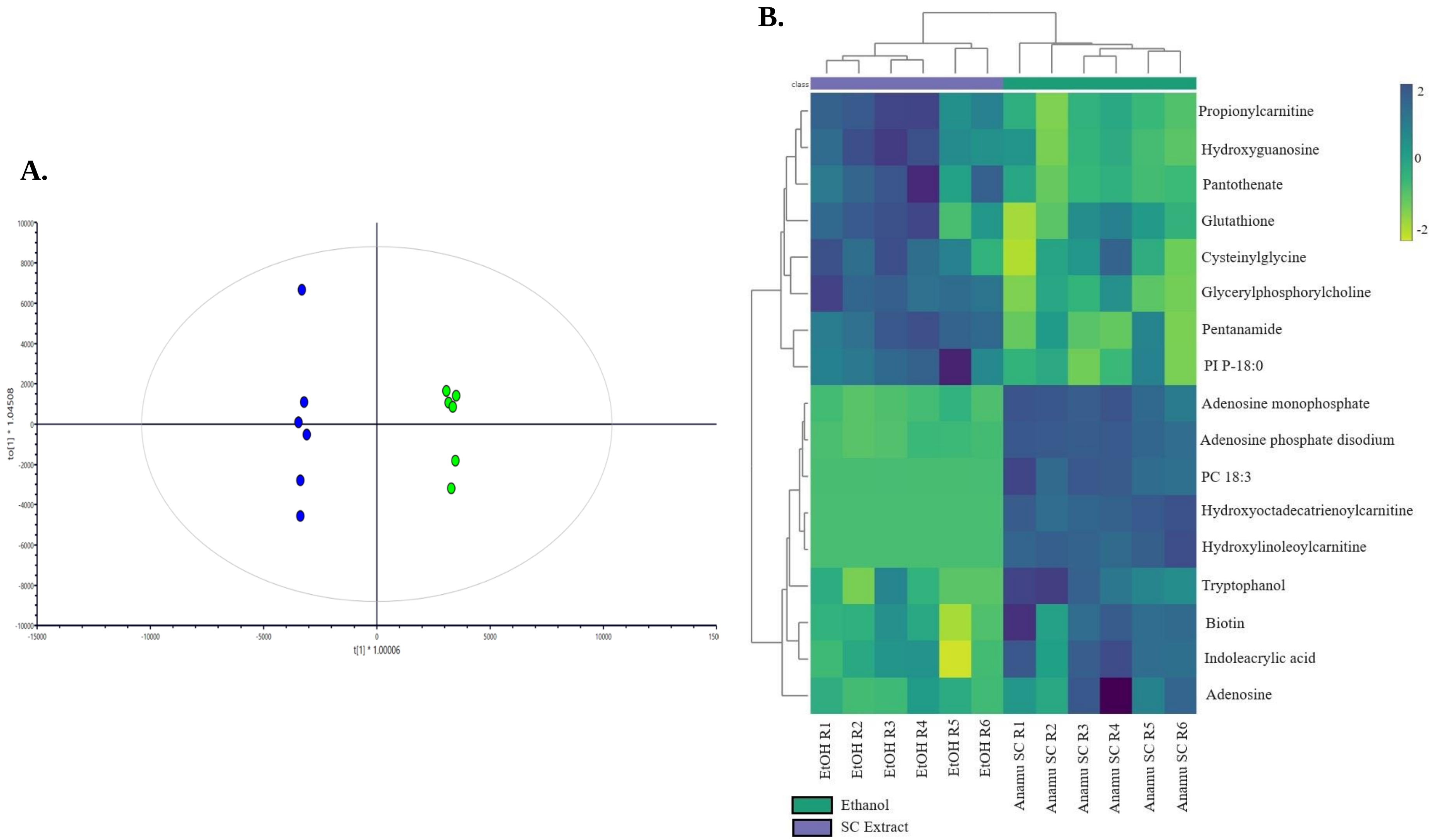

Figure S4. Metabolic differences in K562 cells treated with Anamu-SC vs vehicle, revealed by untargeted metabolome profiling. A. OPLS-DA models with Log transformation, Pareto scaling for metabolic of cell lysate of Anamu-SC extract treatment (green dots), and vehicle (blue dots) groups by LC/MS-QTOF (+) R^2= 0.740, Q^2= 0.981. **B.** Heatmap of the metabolites with statistically significant variation. Upregulated metabolites: blue color, underregulated metabolites: green color.

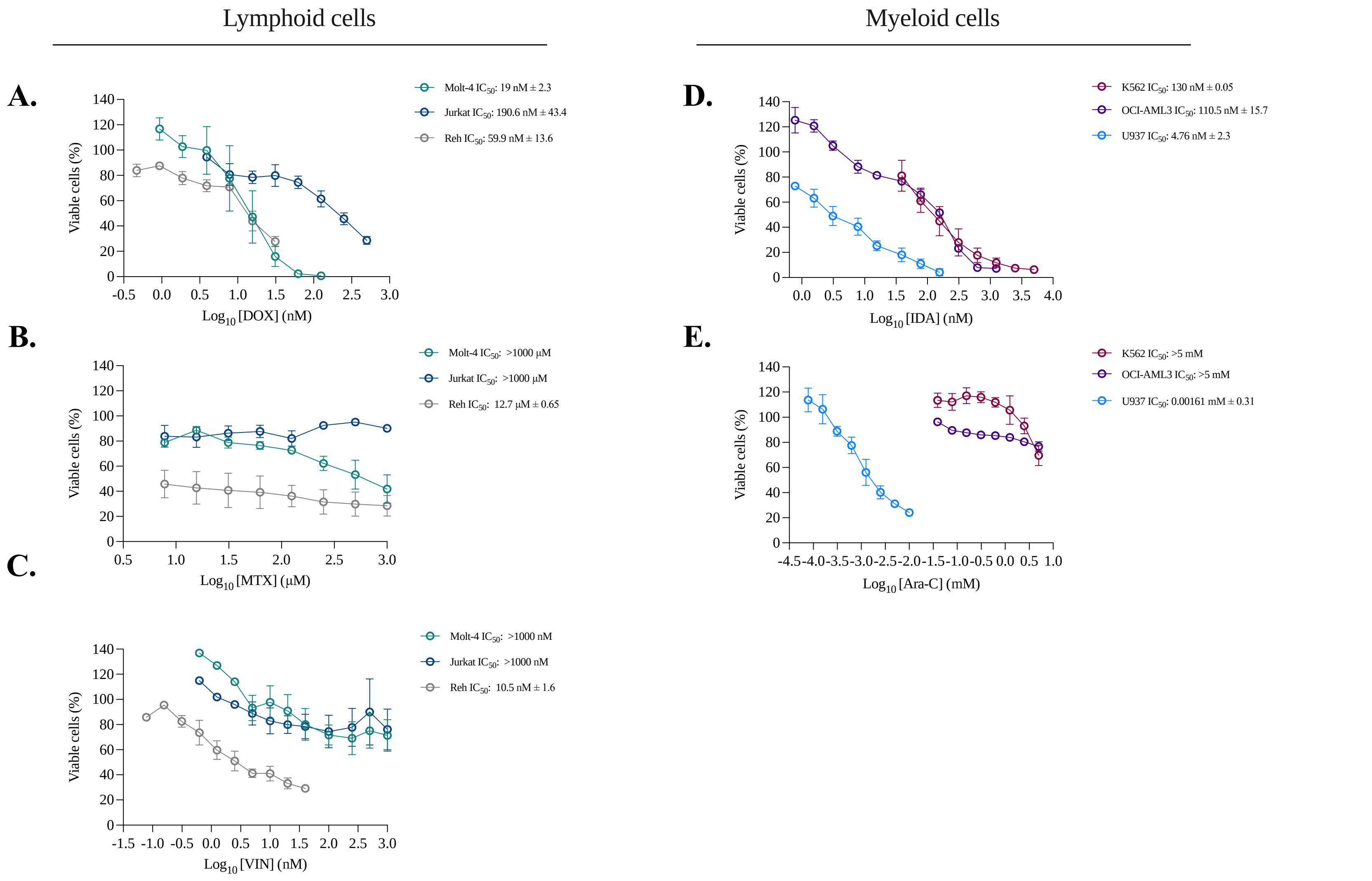

Figure S5. Evaluation of the cytotoxic activity of chemotherapeutic drugs on leukemic lines. Left panels: lymphoid cells; right panels: myeloid cells. **A.** DOX. **B.** MTX. **C.** VIN. **D.** IDA. **E.** Ara-C. Data are represented as the mean ± SD for three independent experiments.

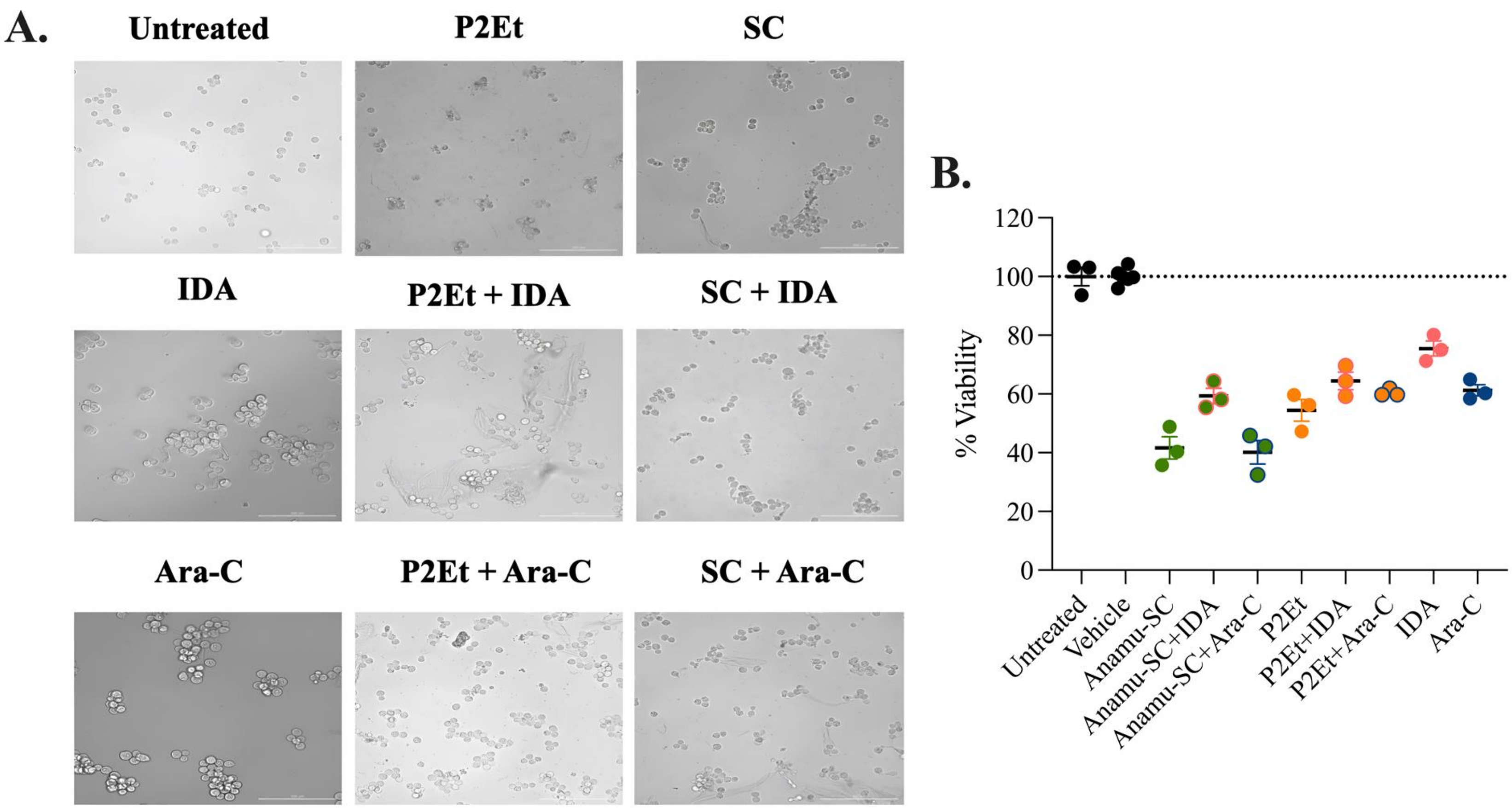

Figure S6. Assessment of the K562 cells' viability was conducted by treating them for 24 h with the extracts and then for 12 h with the chemotherapy. A. Images showing the cells. **B.** Modifications to cell viability. Ara-C: Cytarabine; IDA: Idarubicine.

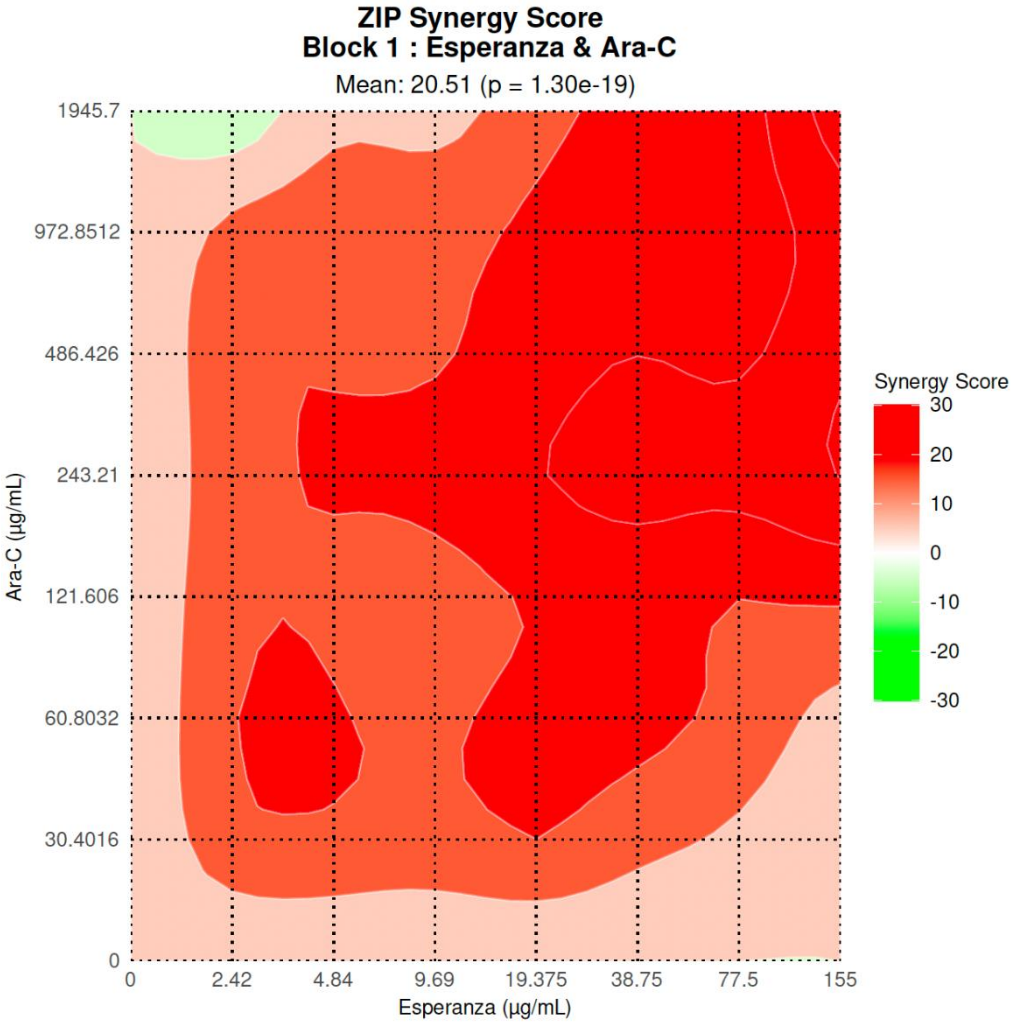

Figure S7. 2D graph prepared by the SynergyFinder + software that shows the synergistic effect between Ara-C and the Esperanza extract on the K562 line.
